# Non-specific adhesive forces between filaments and membraneless organelles

**DOI:** 10.1038/s41567-022-01537-8

**Published:** 2022-03-24

**Authors:** Thomas J. Böddeker, Kathryn A. Rosowski, Doris Berchtold, Leonidas Emmanouilidis, Yaning Han, Frédéric H. T. Allain, Robert W. Style, Lucas Pelkmans, Eric R. Dufresne

**Affiliations:** 1 Department of Materials, ETH Zurich, Zurich, Switzerland; 2 Department of Molecular Life Sciences, University of Zurich, Zurich, Switzerland; 3 Institute of Biochemistry, ETH Zurich, Zurich, Switzerland

**Keywords:** Biopolymers in vivo, Wetting, Polymers, Colloids, Nanoscale biophysics

## Abstract

Many membraneless organelles are liquid-like domains that form inside the active, viscoelastic environment of living cells through phase separation. To investigate the potential coupling of phase separation with the cytoskeleton, we quantify the structural correlations of membraneless organelles (stress granules) and cytoskeletal filaments (microtubules) in a human-derived epithelial cell line. We find that microtubule networks are substantially denser in the vicinity of stress granules. When microtubules are depolymerized, the sub-units localize near the surface of the stress granules. We interpret these data using a thermodynamic model of partitioning of particles to the surface and bulk of the droplets. In this framework, our data are consistent with a weak (≲*k*_B_*T*) affinity of the microtubule sub-units for stress granule interfaces. As microtubules polymerize, their interfacial affinity increases, providing sufficient adhesion to deform droplets and/or the network. Our work suggests that proteins and other objects in the cell have a non-specific affinity for droplet interfaces that increases with the contact area and becomes most apparent when they have no preference for the interior of a droplet over the rest of the cytoplasm. We validate this basic physical phenomenon in vitro through the interaction of a simple protein–RNA condensate with microtubules.

## Abstract (web summary)

Many organelles in the cell are not encapsulated in a membrane—they are liquid-like domains formed through phase separation. The liquid-like nature of such domains leads to adhesive interactions between the cytoskeleton filaments and organelles.

## Main

Phase separation is a physical mechanism utilized by cells to rapidly alter their biochemical landscape by locally concentrating or segregating key molecules^1,2^. The resulting domains are called membraneless organelles and are involved in various processes inside the cell. Typically composed of protein and RNA, they include, for example, nucleoli in the nucleus as well as p-bodies and stress granules in the cytoplasm^3,4^. Membraneless organelles can be very dynamic, exhibiting liquid behaviour such as coalescence^5–8^ and recovery from photobleaching^4,5,9^. In recent years, biologists, chemists and physicists have come together to understand the interplay of phase separation, composition and function of these droplet-like domains^10^.

Phase separation inside cells takes place in an active, viscoelastic environment. Be it the chromatin network of the nucleus or the cytoskeleton of the cytoplasm, protein–messenger ribonucleic acid (mRNA) droplets grow within filamentous networks^11^. Recent studies have focused on the impact of networks on the growth of droplets at scales well beyond the mesh scale. In that case, elastic energy stored in network deformations has been found to substantially alter droplet growth and coarsening, both for synthetic mixtures of oil in cross-linked silicone^12–14^ and protein droplets in chromatin^15–18^.

In the cytoplasm, membraneless organelles interact with filaments of the cytoskeleton. For example, multiple lines of cell biological evidence show that stress granules can interact with microtubules directly or through interaction with microtubule-binding p-bodies^5,19^. Stress granules are liquid-like complexes of proteins and mRNA^20^ that form throughout the cytosol under conditions of biological stress (for example, heat shock or exposure to arsenite), which modulate the translation of cytoplasmic mRNA^4,9,21,22^. As biological stress persists, these granules grow and coalesce to reach a size of up to a few micrometres^23,24^. Microtubules have been suggested to aid stress granule formation by acting as tracks for the active transport of granule components^25–28^ and by encouraging droplet fusion^23,24,29^. Since the characteristic mesh size of the microtubule network is about a micrometre, the dimension of membraneless organelles and microtubule mesh are comparable, and their interactions fall in an experimentally unexplored physical regime. Recent theoretical works suggest that structure and mechanical properties at the pore scale can play an essential role^30,31^.

Here we investigate the physical mechanisms underlying the interaction of microtubules and stress granules. An analysis of their structural correlations shows that the microtubule network densities are enhanced at the surface of the stress granules and decay to their usual value over distances much larger than the granule size. Conversely, large stress granules tend to conform to irregular gaps in the network of microtubules. When microtubules are perturbed by nocadozole, which leads to microtubule depolymerization, tubulin intensities remain enhanced, but more strongly localized, at the surface of the stress granules. These observations point to the adhesive interactions of tubulin and stress granules. A simple and generic model shows that surface adsorption requires no surface-specific molecular functionalization. Instead, it naturally emerges when macromolecular sub-units, with little preference for either phase, assemble into larger structures. Although these non-specific adsorption energies are very small (≲0.1*k*_B_*T*, where *k*_B_ is the Boltzmann constant and *T* is the temperature of the system) for a single tubulin sub-unit, they are sufficient to cause adhesion between microtubules and the surface of stress granules with an adhesion energy of roughly 50*k*_B_*T* μm^–1^. We demonstrate this effect in vitro by comparing the interaction of RNA–protein condensates with tubulin sub-units or microtubules.

To reveal the interactions of membraneless organelles and cytoskeletal filaments, we chose to work with stress granules in U2OS epithelial cells. On fibronectin-functionalized substrates, U2OS spread thoroughly, facilitating cytoskeletal imaging^32^. The formation of stress granules is readily triggered by the addition of small quantities of arsenite to the imaging media^5,21^. An example of the nucleation and growth of stress granules in a live cell tagged for G3BP1 (a stress granule marker protein) and tubulin is shown in Supplementary Video 1 (Supplementary Section 1). This video shows a rich interplay between the microtubules and stress granules, which not only varies over time as the granules grow but also across different positions in the cell. To quantify these interactions, we record the confocal stacks of fixed cells after 90 min of arsenite treatment. We find that granules form within about 15–30 min after treatment, continue to coalesce and grow but show little change in size after about 60 min of treatment. To limit spatial variations, we controlled the cell shapes by plating them on patterned fibronectin patches^32^. All the cells whose final shape did not conform to the pattern were excluded from the analysis. We stained cells for G3BP1 and β-tubulin and acquired stacks of *x*–*y* images separated by Δ*z* = 0.2 μm with a spinning-disc confocal microscope (Nikon Ti2 with Yokogawa CSU-W1; ×100 and numerical aperture (NA) of 1.45).

**Fig. 1:**
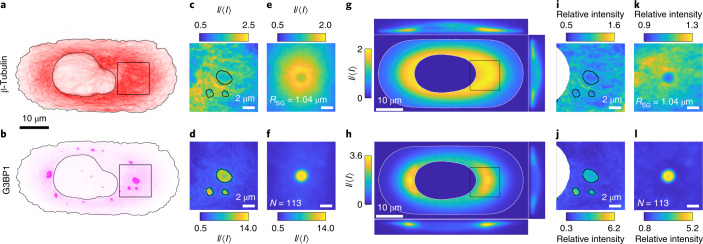
Stress granules and the surrounding microtubule network. **a**,**b**, Examples of *x*–*y* slices of the β-tubulin (**a**) and G3BP1 (**b**) channels of the same cell after 90 min of arsenite treatment. The black line shows the outline of the detected cell and nucleus. **c**,**d**, Images of the stress granule and surrounding microtubule network at the location indicated by the box in **a** (**c**) and **b** (**d**). 〈*I*〉 gives the average intensity of the respective channel across a cell (‘Intensity normalization’ section). **e**,**f**, Averaged images of *N* = 113 stress granules (**f**) and their microtubule network (**e**) for granules with a radius within the interval of 0.98–1.11 μm. **g**,**h**, Examples of *x*–*y* slices of the reference cells calculated from *N* = 335 cells for the β-tubulin (**g**) and G3BP1 (**h**) channels, as well as side views along the respective centre line. The white line indicates the average cell and nucleus shape. The *z* axis has been scaled to match the resolution in the *x*–*y* plane. **i**,**j**, The same images as **c** (**i**) and **d** (**j**) normalized by the intensities of the corresponding location in the respective reference cell (indicated by the black boxes). The intensity is now given in units of relative intensity, where a value of one corresponds to ‘as expected in the reference cell’. **k**,**l**, Distribution maps of β-tubulin (**k**) and G3BP1 (**l**) for granules with a radius within the interval of 0.98–1.11 μm. Source data

Examples of the *x*–*y* slices of G3BP1 and β-tubulin channels 90 min after arsenite treatment are shown in Fig. 1a,b. In this example, we notice several important features. Stress granules have a wide range of sizes, with compact irregular shapes. They tend to be found in the region near the nucleus, which is also rich in microtubules. To quantify these observations, we automatically detect individual stress granules and record their spatial coordinates, shape and size (‘Cell detection and sorting’ section). We find a size distribution of granule radii with a broad peak between 0.5 and 1.0 μm, which decays towards larger sizes with practically no granules larger than 1.5 μm (Supplementary Fig. 1). We classify the granule shape as round or elongated. Round granules have an aspect ratio between 1.0 and 1.5, and make up 79% of the observed stress granules. Elongated granules have aspect ratios greater than 1.5, and can reach values as large as 5.2 (Supplementary Fig. 1). The live-cell imaging of green fluorescent protein (GFP)-G3BP1-tagged stress granules confirms that these large aspect ratios can relax over time and are therefore not a sign of granule hardening (Supplementary Video 1).

To reveal the interactions of stress granules and microtubules, we quantify the structure of the microtubule network in the presence of stress granules. The basis of this analysis is the pairs of images of the G3BP1 and β-tubulin channels centred on a given granule. An example of such an image pair is shown in Fig. 1c,d. For the moment, we consider only round granules with radii greater than 195 nm. This corresponds to the diffraction limit of our optical setup (‘Cell imaging’ section). To visualize the typical shape of both granules and their surrounding network, we average the granule-centred images of both channels for granules of similar radii. Example average images for granules with effective radii of about 1.04 μm are shown in Fig. 1e,f. The average images for all the size bins are provided in Supplementary Figs. 2 and 3.

The average image of the stress granule channel shows a circular granule with an intensity greater than ten times the average G3BP1 intensity in the cell. The average β-tubulin images reveal a volcano-like shape, with a central region of relatively lower intensity that matches the size and shape of the granule. This circular region is surrounded by a ring-like structure of almost twice the normal tubulin intensity, followed by a decay in intensity over several micrometres towards its average value.

G3BP1 and tubulin are not homogeneously distributed in the cell. To quantify how local perturbations of the microtubule network are correlated with the presence of stress granules, we, therefore, needed to account for systematic variations in the microtubule architecture and G3BP1 concentration across the cell. To do so, we constructed ‘reference cells’, spatially resolved image stacks for each channel averaged across the full ensemble of cells. Details of the image alignment, construction and normalization are described in the ‘Construction of the reference cell’ section. The reference cells provide the expected intensity of the β-tubulin and G3BP1 channels for each three-dimensional location in the cell, as well as the average cell shape and location of the nucleus. Slices of G3BP1 and β-tubulin reference cells in the *x*–*y*, *x*–*z* and *y*–*z* planes are shown in Fig. 1g,h. Both tubulin and G3BP1 are the brightest near the nucleus.

With the reference cells in place, we calculate the enhancement of G3BP1 and β-tubulin intensity in and around the individual granules by a point-wise division of the stress granule or β-tubulin images by the intensity of the corresponding reference cell at the same location. Figure 1i,j shows the same images as Fig. 1c,d, where each pixel is now normalized by the respective reference. These normalized images are now given in the dimensionless unit ‘relative intensity’, where a value greater (less) than one indicates that the intensity is higher (lower) than expected at that location. For example, a relative intensity of 1.2 of tubulin in the vicinity of a granule denotes a 20% increase in tubulin intensity compared with the ensemble average at that location. Note that pixels that are masked due to being outside the cell or inside the nucleus in either an individual image and/or the reference are discarded.

Averaging these normalized images for the same granule size and condition, we arrive at distribution maps (Fig. 1k,l). Qualitatively, the average images (Fig. 1e) and distribution maps for the G3BP1 channel are fairly similar. The normalization largely just rescales the relative intensities. For the tubulin channel, however, normalization by the local intensity has a stronger effect. It flattens the intensity distribution outside the stress granule, while retaining the enhancement of tubulin around its surface. Within the granule, the tubulin signal drops to a value close to one.

**Fig. 2:**
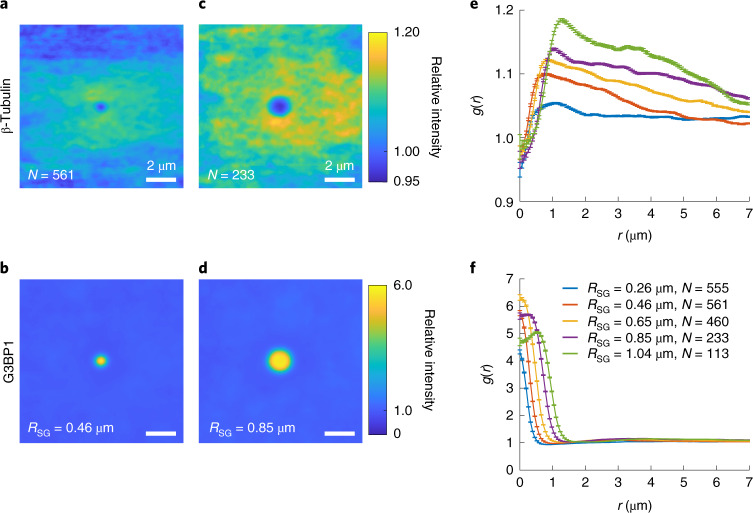
Microtubule network is modulated in the presence of stress granules. **a**, Distribution map of the β-tubulin channel for round granules with radius *R*_SG_ ranging from 0.39 μm to 0.52 μm. *N* gives the number of contributing images. **b**, Corresponding distribution map for G3BP1. **c**, Distribution map of the β-tubulin channel for round granules with a radius ranging from 0.78 μm to 0.91 μm. **d**, Corresponding distribution map for G3BP1. **e**, Radial distribution function *g*(*r*) of β-tubulin around round granules of different sizes; error bars show the standard error. **f**, Corresponding *g*(*r*) of G3BP1.

These basic features are found for granules of all the sizes. However, the enhancement of the microtubule signal around the stress granules is stronger for larger granules (Fig. 2a–d). For a quantitative comparison, we azimuthally average the distribution maps, forming a radial distribution function *g*(*r*) for the tubulin and stress granule channels. Note that this *g*(*r*) is similar, but not identical, to the classic radial distribution function employed across condensed-matter physics. These curves are shown for various granule radii (Fig. 2e,f). As expected for a compact phase-separated object, *g*(*r*) for G3BP1 peaks near *r* = 0 and decays to one over a distance corresponding to the granule radius. For β-tubulin, *g*(*r*) increases from a value near one at the centre of the granule (*r* = 0) to a peak just outside the granule, followed by a slow monotonic decay over a distance much larger than the size of the granule.

We validated this novel method to characterize the structural correlations in a heterogeneous environment with a computational negative control. To construct the distribution maps with no structural correlations, we used the measured position and size of each stress granule, but extracted the images of the G3BP1 and tubulin channels at the same location in a different cell. As expected, the resulting distribution maps for both G3BP1 and β-tubulin showed none of the features described above, but simply fluctuate around a value of one (Supplementary Figs. 2 and 3).

**Fig. 3:**
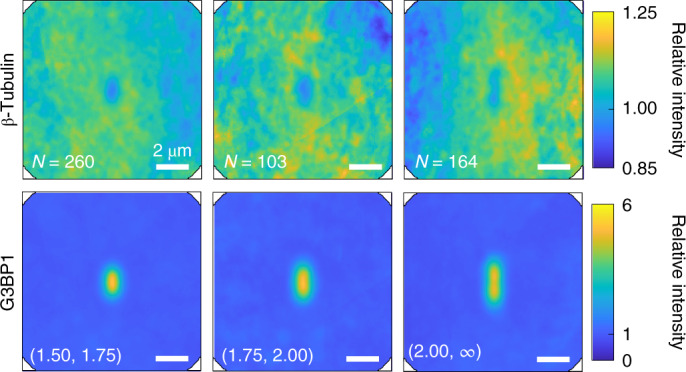
Elliptical granules correlate with elliptical gaps in the microtubule network. Distribution map of the tubulin network and the corresponding granules binned by the principal axis ratio. The area of all the granules is between 0.5 μm^2^ and 1.5 μm^2^. Both individual granules and tubulin images are rotated such that the long axis of each granule is vertical.

So far, we have considered only granules with a principal axis ratio of up to 1.5. Now, we turn our attention to the network around elongated granules. Figure 3 shows the distribution maps for granules with larger ellipticity. Here we keep the size of the granule constant and bin the granules by their principal axis ratio. The construction is analogous to the distribution maps of round granules; only now, the individual images of granules, as well as the corresponding image of the tubulin channel, are rotated such that the major principal axis is vertical before averaging. We find that elongated granules are correlated with complementary depletions in the β-tubulin intensity that match their size and shape. On average, larger stress granules are more elongated, suggesting that larger granules are more affected by the network (Supplementary Fig. 1h). Since the favoured shape of a liquid droplet is spherical, elongated droplets reflect anisotropic forces acting on them. The correspondence in shape of the granules and the ‘cavity’ in the tubulin channel suggests that microtubule networks can deform stress granules.

Our results suggest that microtubule networks are deformed in the presence of stress granules (Fig. 2) and that stress granules conform to the local structure of the microtubule network (Fig. 3). To perturb microtubules, we treat cells with 0.5 μg ml^–1^ (1.67 μM) nocodazole. Nocodazole hinders microtubule polymerization, leading to the depolymerization of the network within 10–20 min^33^. Cells are treated for 90 min with both arsenite and nocodazole before fixation. After nocodazole treatment, filamentous structures are absent from the β-tubulin channel, and stress granules are smaller and more numerous (Supplementary Fig. 1e), in line with previous observations^23^. Note that we cannot confirm that microtubules are fully depolymerized into sub-units; tubulin may also be present in the form of small aggregates below the optical resolution.

**Fig. 4:**
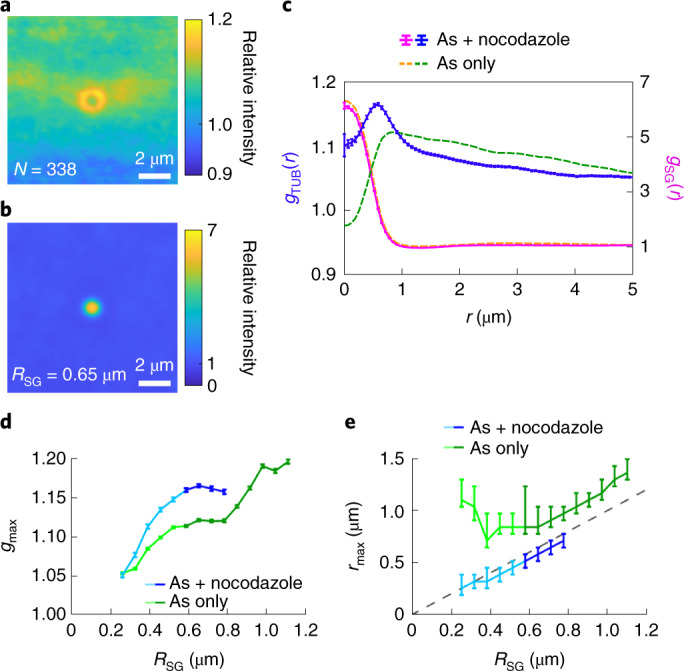
β-Tubulin localizes at the granule surface in nocodazole-treated cells. **a**,**b**, Distribution map of β-tubulin (**a**) and G3BP1 (**b**) for granules with a radius ranging from 0.59 μm to 0.72 μm in nocodazole-treated cells. **c**, *g*(*r*) for the distribution maps of β-tubulin in **a** (*g*_TUB_(*r*)) and G3BP1 in **b** (*g*_SG_(*r*)) (solid line; error bars show the standard error) compared with the respective *g*(*r*) of the same size of granules with an intact microtubule network (dashed lines). **d**, Maximum value, *g*_max_, of the β-tubulin *g*(*r*) for granules of different radii calculated as the running average considering data for the indicated radius ±0.065 μm. The error bars show the standard error. The data are shown for both nocodazole-treated cells and cells with arsenite treatment only. The darker colours indicate the size of the granules where the intensity of G3BP1 plateaus inside the granule. **e**, Running average of the position of the maximum value *r*_max_ in the β-tubulin *g*(*r*) for granules of different radii in nocodazole-treated cells and those treated with arsenite only. The dashed line indicates the detected granule surface. The error bars are given as the interval of granule radii, where the corresponding *g*(*r*) falls within the error of *g*_max_.

Distribution maps of nocodazole-treated cells are shown in Fig. 4a,b. The distribution map of β-tubulin shows a relatively narrow ring surrounding the peak in the G3BP1 channel. This observation becomes clearer in *g*(*r*), which reveals a peak localized at the granule surface. A direct comparison of the *g*(*r*) values of β-tubulin and G3BP1 (Fig. 4c) reveals that the surface enhancement of β-tubulin is more localized in nocodazole-treated cells. Further, the maximum value of the β-tubulin *g*(*r*) is typically higher in nocodazole-treated cells for granules of all sizes (Fig. 4d). Finally, the location of the β-tubulin peak, *r*_max_, is consistently closer to the centre of the granule in nocodazole-treated cells (Fig. 4e). Distribution maps of stress granules of various sizes and the corresponding tubulin channel for nocodazole-treated cells, as well as the relevant negative controls, are shown in Supplementary Figs. 4 and 5.

**Fig. 5:**
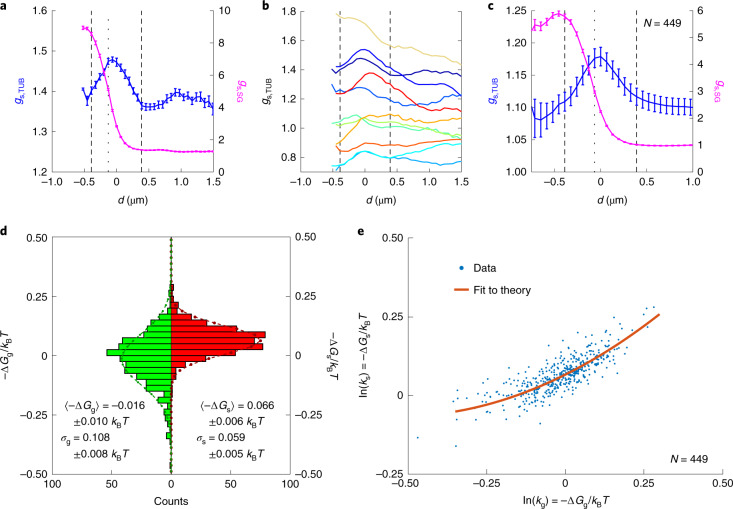
Tubulin sub-units in nocodazole-treated cells adhere to the granule surface. **a**, Surface-relative distribution function *g*_s_(*d*) for β-tubulin (*g*_s,TUB_(*d*)) and G3BP1 (*g*_s,SG_(*d*)) channels for a single stress granule in a cell treated with nocodazole. The dashed vertical lines at ±0.39 μm indicate the approximate width of surface enhancement. The dotted vertical line shows the position of the maximal slope in the G3BP1 channel. The error bars indicate the standard error. An image of this granule and surrounding tubulin is shown in Supplementary Fig. 6. **b**, Values of *g*_s_(*d*) for the β-tubulin channel for randomly chosen granules. The error bars are of the same magnitude as in **a**. **c**, Averaged *g*_s_(*d*) of the tubulin and G3BP1 channels for all sufficiently large granules (*N* = 449), that is, collected over all the data shown in the scatter plot in **e**. The dashed lines show the assumed width of the interface at ±0.39 μm and the dotted line shows the position of the maximal gradient in *g*_s,SG_(*d*). The error bars indicate the standard error. **d**, Histogram of the natural logarithm of the bulk partitioning coefficient ln(*k*_g_) = –Δ*G*_g_ and the surface partitioning ln(*k*_s_) = –Δ*G*_s_. The dashed lines indicate a Gaussian fit to each histogram with the respective mean affinities 〈–Δ*G*〉 and variance *σ*. **e**, Scatter plot of ln(*k*_s_) as a function of ln(*k*_g_). Each point corresponds to one granule (*N* = 449). The red line shows the best fit of the theory to the data. The mean errors of individual data points are ±0.013*k*_B_*T* for ln(*k*_s_) and ±0.008*k*_B_*T* for ln(*k*_g_). The data in **e** with error bars are shown in Supplementary Fig. 7.

The persistent preference of tubulin, with or without a well-defined microtubule network, for the surface of stress granules suggests that tubulin may have an affinity for the surface of stress granules. To investigate this more precisely, we quantify the intensities as a function of distance *d* from the stress granule surface, instead of distance *r* from the centre of the granule (‘Correlation analysis’ section). In this way, we can consolidate the data from granules of different sizes and shapes. The interface (*d* = 0) is defined by the granule detection routine. By averaging intensities for pixels with the same *d* from reference-cell-normalized images, we arrive at a surface-relative distribution function, *g*_s_(*d*), for each granule. An example of *g*_s_(*d*) for the G3BP1 and β-tubulin channels of one granule in a nocodazole-treated cell is shown in Fig. 5a. Here *g*_s_(*d*) for the G3BP1 channel reveals an interface region over which the intensity of G3BP1 smoothly transitions from the inside of the granule to the cytosol. The corresponding *g*_s_(*d*) for tubulin has a strong peak within this interface zone. Although there is strong variation in the β-tubulin *g*_s_(*d*) from granule to granule (Fig. 5b), we typically observe a local maximum within this interface zone. This observation becomes clear in the average *g*_s_(*d*) of β-tubulin, which shows a peak centred within the interface zone of the G3BP1 *g*_s_(*d*) (Fig. 5c). Based on the average *g*_s_(*d*) for G3BP1, we define the interface zone of width ∣*d*∣ < 0.39 μm, where G3BP1 intensities transition from their values in the stress granule to their values in the bulk.

We quantified the affinities of tubulin sub-units for the surface and bulk of stress granules using partition coefficients. Generally speaking, partition coefficients measure the concentration of a species in a particular domain relative to their values in a nearby reservoir (here the cytosol). We defined the surface partition coefficient *k*_s_ as the ratio of the peak value of *g*_s,TUB_(*d*) within the interface zone to its value just beyond the interface zone, outside the granule. Similarly, the partition coefficient for the bulk of an individual granule *k*_g_ is the ratio of *g*_s,TUB_(*d*) just inside to that just outside the interface zone (‘Calculation of the partitioning coefficients’ section). Given the broad apparent interface region, we only consider granules with a radius of 0.59 μm and larger, that is, granules with a defined bulk phase where the G3BP1 intensity plateaus inside the granule. Note that these partition coefficients are based on intensities and not concentrations. Therefore, systematic variations in fluorophore efficiency or antibody binding in different environments could lead to systematic errors in partition coefficients. However, we expect such errors to be negligible in the limit of weak bulk partitioning (*k*_g_ ≈ 1) as observed here.

Assuming local thermodynamic equilibrium of tubulin near the surface of the granule (Supplementary Section 3A), we can relate the partition coefficients to the free-energy difference of tubulin relative to the cytosol:

1 $${k}_{{\mathrm{g}},{\mathrm{s}}}=\exp \left(-{{\Delta }}{G}_{{\mathrm{g}},{\mathrm{s}}}/{k}_{\mathrm{B}}T\right).$$

Here Δ*G*_g_ and Δ*G*_s_ indicate the free-energy difference of tubulin inside and on the surface of the stress granules, respectively, relative to the cytosol. Histograms of the bulk and surface affinities of tubulin, namely, −Δ*G*_g_ and −Δ*G*_s_, respectively, are shown in Fig. 5d. We find that the distribution of affinities for the bulk of the granules has a small but statistically significant (*p* = 0.002, one-sample *t*-test) mean of −0.016*k*_B_*T*, suggesting a subtle preference for the cytosol. On the other hand, the distribution of surface affinities has a stronger and more significant (*p* < 0.001) mean value of 0.066*k*_B_*T*. This suggests that tubulin sub-units have weak attraction to the interface of stress granules. The apparent surface and bulk affinities are correlated (Fig. 5e). The surface affinity increases with the bulk affinity, but remains positive as the bulk affinity vanishes.

This behaviour is consistent with a simple physical picture where tubulin is attracted to the surface to reduce the interfacial energy of stress granules. In other words, tubulin acts as a very weak surfactant. To capture the essential physics, we developed minimal physical models where tubulin (in the form of a sub-unit or a small aggregate) has no specific molecular mechanism for attachment to the surface of a stress granule. As in the theory of Pickering emulsions^34–36^, tubulin’s affinity for the bulk of the granule is −Δ*G*_g_ = *−A*_0_Δ*γ* = −*A*_0_(*γ*_tg_ − *γ*_tc_). Here *A*_0_ is the surface area of the tubulin sub-unit, and *γ*_tg_ and *γ*_tc_ are the interfacial energies of tubulin against the granule and cytoplasm, respectively. In the special case where tubulin would have no preference for either phase (−Δ*G*_g_ = 0), it would be bound to the interface with affinity −Δ*G*_s_ =*A*_*x*_*γ*_cg_, where *A*_*x*_ is the area of the interface covered by the tubulin sub-unit and *γ*_cg_ is the interfacial energy between both liquid phases (Supplementary Section 3B). The affinity is simply caused by the reduction in energy of the interface when part of the interface is covered by a particle. When the particle is larger, it takes up more area at the interface and is more strongly bound. Assuming that tubulin attaches to the interface in sub-units with *A*_*x*_ ≈ 25 nm^2^ (ref. ^37^), the observed surface affinity at −Δ*G*_g_ = 0 of about 0.1*k*_B_*T* is consistent with an interfacial energy of the stress granules of *γ*_cg_ ≈ 15 μJ m^–2^, which is well within the range of typical values for membraneless organelles^6,38^. Since a single sub-unit is the smallest plausible aggregate size, this estimate is an upper bound for the interfacial energy.

The theory of Pickering emulsions, however, was developed for systems where the particle size is much larger than the interface thickness. Since tubulin sub-units have a similar size to the components of stress granules, we do not expect it to apply here. Therefore, we extend the basic Pickering concept to situations where particles interact with an interface of finite width *w*. In the limit where the particle size is much smaller than *w*, the free-energy difference between a particle at position *x* and bulk cytosol (*x* → *∞*) has two terms, one proportional to its surface area *A*_0_ and the other to its volume *V*_0_:

2 $${{\Delta }}G(x)={A}_{0}\,(\gamma (x)-\gamma (\infty ))-{V}_{0}\,(f(x)-f(\infty )).$$

The area term captures the interfacial energy between the particle and fluid and the volume term describes the energy of the fluid displaced by the particle. To determine the free energy of the particle localized to a thick interface, we follow the description of Cahn and Hillard comprising a simple two-component system^39–41^. Expanding the energy landscape around the centre of the interface (*x* = 0) and keeping terms up to the second order in *x*/*w*, we find

3 $${{\Delta }}G(x)\approx {A}_{0}\,\frac{{{\Delta }}\gamma }{2}\left(1-\frac{x}{w}\right)-{V}_{0}\,\frac{3}{2}\frac{{\gamma }_{\mathrm{cg}}}{w}\left(\frac{1}{2}-\frac{{x}^{2}}{{w}^{2}}\right).$$

A particle at the interface then has the equilibrium position

4 $$\begin{array}{r}{x}_{\mathrm{eq}}=\frac{{A}_{0}{w}^{2}}{6{V}_{0}}\frac{{{\Delta }}\gamma }{{\gamma }_{\mathrm{cg}}}\end{array}$$

with the corresponding affinity

5 $$-{{\Delta }}{G}_{\mathrm{s}}={a}_{0}+\frac{1}{2}(-{{\Delta }}{G}_{{\mathrm{g}}})+\frac{{(-{{\Delta }}{G}_{{\mathrm{g}}})}^{2}}{32{a}_{0}}.$$

We find a single physical parameter *a*_0_, which gives the surface affinity of a tubulin particle at −Δ*G*_g_ = 0. For a spherical particle with radius *R*

6 $${a}_{0}=\uppi {R}^{2}{\gamma }_{\mathrm{cg}}\left(\frac{R}{w}\right).$$

Note that the thin interface model gives a similar quadratic form with different pre-factors. Complete derivations of each theory are provided in Supplementary Section 3B,C. By fitting the data in Fig. 5e to equation (5) with only one fit parameter, we find *a*_0_ = 0.067 ± 0.002*k*_B_*T* (‘Fitting’ section).

The above analysis of nocodazole-treated cells shows that tubulin fragments have small affinity (≲0.1*k*_B_*T*) towards the surface of stress granules. As tubulin polymerizes to form microtubules, this affinity should linearly increase with the length of the filament. Assuming that the surface affinity measured in nocodazole corresponds to individual sub-units, microtubules as short as 100 nm will bind to the surface of the stress granules with an adhesion energy of more than 5*k*_B_*T*. This implies that microtubules can strongly bind to the surface of stress granules, even when tubulin sub-units do not.

**Fig. 6:**
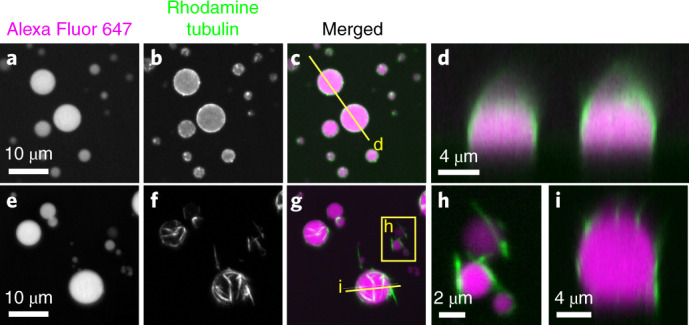
β-Tubulin and microtubules localize to the surface of FUS-RNA droplets in vitro. **a**–**c**, FUS-RNA droplets with tubulin sub-units. The droplet is visualized with Alexa Fluor 647 (**a**) and tubulin with rhodamine (**b**) and the merged data are provided in **c**. The mid-plane of a *z* stack is shown. **d**, Side view of two droplets at the location indicated by the line in **c**. **e**–**g**, FUS-RNA droplets with taxol-stabilized microtubules. The maximum projection along *z* is shown to visualize all the filaments around the droplets. **h**, Zoomed-in image of two small droplets at the location indicated by the box in **g**. **i**, Side view of a droplet at the location indicated by the line in **g**.

To test this, we performed experiments on a simplified system in vitro. In place of stress granules, we made protein–RNA condensates with full-length fused in sarcoma (FUS) protein (a component of stress granules^9^) and RNA (poly-U) (‘Droplets in vitro’ section). In this minimal system, we observed a substantial affinity of rhodamine-labelled tubulin sub-units for the surface of the droplets (Fig. 6a–d). Strikingly, taxol-stabilized microtubules have a much stronger preference for the surface of the droplet (Fig. 6e–i). This is consistent with the simple model proposed here, and is reminiscent of other recent in vitro observations in which filaments were strongly localized to the surface of synthetic phase-separated polymer droplets^42,43^.

Previous experiments and theory on the interaction of phase separation and macromolecular networks have focused on the expansion of cavities in elastic networks^12–15,18,30,44^. These deformations are driven by the free energy per unit volume liberated by condensation, that is, condensation pressure. For a single phase-separating component

7 $${p}_{{{{\mathrm{cond}}}}}={n}_{{\mathrm{L}}}{k}_{\mathrm{B}}T\ln (n/{n}_{{{{\mathrm{sat}}}}}),$$

where *n*_L_, *n* and *n*_sat_ are the number densities in the droplet, at the continuous phase and at the point of saturation in the continuous phase, respectively^12^. When the surrounding medium is an ideal elastic solid, cavities grow without bound when *p*_cond_ > 5*E*/6, where *E* is Young’s modulus of the surrounding medium^44^. Although these ideas are well suited for permanently cross-linked networks, viscoelastic relaxation of the cytoskeleton^45–48^ makes them largely irrelevant to the steady state of stress granules. Indeed, Supplementary Video 1 clearly shows that stress granules and microtubules can rearrange over timescales of a few minutes. Consequently, we expect elastic stresses induced by droplet growth to be fully relaxed within 90 min after the induction of granules—the focus of the current study.

Instead, the adhesion of tubulin and stress granules, as described above, appears to be the driving force underpinning the observed structural correlations of stress granules and tubulin. Generically, these surface affinities can do work when the adhesion energy is positive:

8 $$W={\gamma }_{\mathrm{cg}}+{\gamma }_{\mathrm{tc}}-{\gamma }_{\mathrm{tg}} > 0.$$

Since the results for nocodazole-treated cells are consistent with ∣*γ*_tc_ − *γ*_tg_∣ ≪ *γ*_cg_, we expect *W* ≈ *γ*_cg_. This positive adhesion energy could drive the deformation of the microtubule network (Fig. 2) or stress granules (Fig. 3). Since the microtubule network presents little resistance to deformation on long timescales, we expect adhesive forces to consolidate the microtubule network around the droplet. This is consistent with macroscopic experiments and theory, showing that neutrally wetting droplets preferentially sit at the side of filaments^49^. Because the persistence length of microtubules is large, concentration enhancements around the stress granules must decay slowly (Fig. 2). Note that microtubules are too rigid to be substantially bent by sub-micrometre stress granules^50–53^ (Supplementary Section 4), whereas some bending is apparent in the adhesion of microtubules to larger in vitro droplets (Fig. 6g and Supplementary Fig. 8). Since the contact area of microtubules and stress granules increases with the size of the granule, we expect stronger adhesion for larger granules (Fig. 2). Over long timescales, adhesion, thus, favours the migration of stress granules to regions of the cell with higher microtubule concentrations. In our experiments, we find that the microtubule-rich regions on either side of the nucleus are also the preferred location of stress granules (Fig. 1g,h). This effect is quantified in Supplementary Fig. 9, where a voxel-wise cross-correlation of tubulin and G3BP1 intensities shows a strong tendency for granules to be found in regions of above-average tubulin intensity.

We have introduced a statistical method to quantify the structural correlations of various components in living cells, and identified substantial interactions of microtubules and stress granules. This approach should be applicable to quantify a variety of weakly bound structures within the cell. Here the depolymerization of microtubules and a physical model of adsorption support the hypothesis that non-specific adhesive interactions are sufficient to drive the observed structures. Tubulin sub-units’ weak affinity for the surface of stress granules, as well as weak repulsion from the bulk of stress granules, are amplified as microtubules polymerize, leading to the distinct enhancement of tubulin density around granules. With more precise structural data on the thickness of the interface between membraneless organelles as well as on the size of adsorbing particles, non-specific adsorption could provide a route to measure in vivo surface tension through the fit parameter *a*_0_.

These interfacial phenomena could impact a wide range of cellular phenomena. Surface partitioning has recently been suggested to play a role in the regulation of P granules^54^ and could contribute to the observed core–shell structure of stress granules^9^. Wetting interactions of protein droplets with microtubules have been shown to facilitate branching^55,56^. Because of their non-specific nature, we expect that interfacial forces could play a role in a number of interactions of membraneless organelles with other supramolecular structures. For example, the interaction of phase-separated domains with membrane structures including the endoplasmic reticulum^57^, phagosome^58^, Golgi apparatus^59^ and synaptic vesicles^60^. We anticipate that similar interactions may also impact the localization of pathological aggregates as observed during neurodegenerative diseases^2,61^ or of functional aggregates in the formation of the skin barrier^62,63^.

## Methods

### Materials and methods

#### Cell culture

U2OS human osteosarcoma cells were grown in Dulbecco’s modified eagle medium (DMEM) supplemented with 10% fetal bovine serum (ThermoFisher) and 2 mM l-glutamine (ThermoFisher) at 37 °C in 5% CO_2_. For the experiments, patterned coverslips were coated with 20 μg ml^–1^ fibronectin (Sigma-Aldrich), and single cells were plated on these coverslips 4–6 h before the experiments to ensure sufficient spreading.

#### Micropatterning

Glass coverslips were incubated in 0.1 mg ml^–1^ poly-l-lysine-*g*-poly(ethyleneglycol) (PLL(20)-g[3.5]-PEG(2), SuSoS AG) for 1 h. The coated coverslips were then exposed to deep ultraviolet light coated-side up in an ultraviolet/ozone cleaner (ProCleaner Plus, BioForce Nanosciences) through a chrome-on-quartz photomask (printed by Deltamasks). The transparent features on the otherwise opaque photomask are 25 μm × 30 μm rectangles with two hemispherical caps of radius 12.5 μm at the ends and several hundred features per coverslip. The features are spaced at least 100 μm apart from edge to edge in all directions. After ultraviolet treatment, the patterned coverslips were coated with fibronectin and the cells were plated.

#### Live-cell imaging

Single U2OS cells stably expressing GFP-tagged G3BP1 (Pelkmans Lab^64^) were plated on patterned coverslips following the protocol mentioned above. After spreading for 3–4 h, the cells were switched into media containing 100 nM SiR-tubulin (Spirochrome) and 10 μM verapamil (Spirochrome) and incubated in the dye for at least 4 h. The cells were imaged at 37 °C in imaging media containing Leibovitz’s L-15 medium (ThermoFisher Scientific) with 10% fetal bovine serum, 30 μl ml^–1^ Oxyrase (Sigma-Aldrich) and 10 mM lactic acid, with 0.5 mM sodium arsenite (Sigma-Aldrich). Imaging was done on a Nikon Ti2 Eclipse epifluorescent microscope with a cage incubator (Okolab), using ×100 oil objective with numerical aperture (NA) of 1.49 and a Nikon DS-Qi2 camera.

#### Immunofluorescence

After specified durations of treatment with 0.5 mM sodium arsenite (Sigma-Aldrich) to induce stress granule formation^5^, wild-type U2OS cells were fixed with 4% formaldehyde for 15 min, permeabilized with 0.1% Triton X-100 in phosphate-buffered saline (PBS) and blocked with 5 mg ml^–1^ bovine serum albumin (Sigma-Aldrich), in some cases also containing 0.25% Triton X-100. Fixed cells were incubated overnight at 4 °C with primary antibodies (mouse anti-G3BP (1:500, abcam ab56574) and rabbit anti-β-tubulin (1:200, abcam ab6046)) to stain the stress granules and microtubules. Note that G3BP does not co-precipitate in β-tubulin immunoprecipitation and is commonly used as a stress granule marker^26,65^. Secondary antibodies consisted of rhodamine Red-X anti-mouse IgG (1:500, Jackson ImmunoResearch) and Alexa Fluor 647 anti-rabbit IgG (1:500, Jackson ImmunoResearch). DNA was stained by incubating in 4′,6-diamidino-2-phenylindole (DAPI) (1:500, Sigma) for 15 min at room temperature. Coverslips were mounted in ProLong Gold (ThermoFisher).

#### Cell imaging

Fixed cells were imaged on a Nikon Ti2 Eclipse with Yokogawa CSU-W1 spinning disc and 3i 3iL35 LaserStack using ×100 oil objective with NA of 1.45 and a Hamamatsu ORCA-Flash4.0 camera. The spatial resolution in the focal (*x*–*y*) plane was 0.065 μm per pixel and the step height (*z* axis) was 0.200 μm. The theoretical diffraction limit for the shortest used wavelength (488 nm) is 130 nm, calculated as $\frac{0.5\lambda }{\sqrt{2}{\mathrm{NA}}}$, that is, the full-width at half-maximum of the theoretical point spread function of a confocal microscope^66^. For the actual optical setup, we assume a diffraction limit of 3 pixels (195 nm).

#### Cell detection and sorting

Image analysis has been carried out using MATLAB version R2020b and has been automated to allow for the efficient and reproducible processing of a large number of cells. Each set of confocal data acquired on the microscope contained one cell. The cell has been identified in the *x*–*y* plane using the regionprops function (MATLAB) on an overlay of the maximum projections along the *z* direction of all the channels as well as a wide-field image of the nucleus through the DAPI stain. For the accurate detection of the cell shape in the focal plane, the image has been sharpened before thresholding. All the images have been cropped around the detected cell in the *x*–*y* plane. To determine the *z* coordinate of the base of each cell (cell base height), the sum of the median and 80th percentile of each *x*–*y* plane for the filament channel has been collected. With only a small rim of background around the cropped cells, the median of the image is slightly below the median intensity of the cell and serves as a proxy for the overall structure of the cell. The 80th percentile captures more pronounced features, such as individual filaments, while being robust against outliers. Then, the cell base height has been calculated as the *z* coordinate corresponding to the maximum gradient of this intensity measure, which reliably detected the side of the cell attached to the coverslip. We assume the error of this measurement to be ±1 *z* step. The maximum intensity of the *z* slice was typically 2–4 slices above the cell base height. Cells in which this distance fell out of this interval were discarded. Moreover, the cells with an area outside 85–110% of the area of the prescribed pattern (1,257 μm^2^) were discarded. Cells with a ratio of the short principal axis to the long principal axis outside the interval [0.43, 0.55] were also discarded. The corresponding ratio of the pattern itself is 0.45. These criteria have been chosen based on the corresponding histograms of all the cells to discard outliers.

The background intensity of each channel has been approximated from the intensity in the corners of the cropped image outside the detected cell. Each channel has been individually corrected for this background intensity.

Multiple cells were recorded on each coverslip in one acquisition session. Within one such batch, all the patterns have the same orientation. The orientation of the patterns has been determined as the mean orientation of the cells from one batch. Batches with less than 10 cells remaining after filtering the cell area and shape have been discarded. Individual cells with an orientation that deviates by more than 3° relative to the pattern orientation were also discarded. All the cells have been rotated by the mean orientation of the pattern to ensure consistent cell orientation across the samples. The remaining cells were aligned such that the centroid of the cell was in the centre of the *x*–*y* plane. Because the nucleus is not always exactly in the centre of the cell, the cells were arranged such that the centroid of the nucleus always falls in the same hemisphere of the image, that is, some cells were rotated by 180°. This yields cell stacks that are oriented in the *x*–*y* plane such that the long axis of the cell is horizontal and the nucleus falls to the left side.

#### Intensity normalization

The intensities of the G3BP1 and β-tubulin channels of each cell were individually normalized by their mean intensity 〈*I*〉 to account for differences in protein expression or staining. To define this mean intensity, we select a set of representative pixels. The *x* and *y* coordinates of the representative pixels are those that fall inside the cell shape but outside the cell nucleus, based on the maximum projection along the *z* coordinate of all the channels. The pixels outside the cell or inside the nucleus were set to a value of NaN (not a number) throughout the subsequent analysis. The *z* coordinates of the representative pixels are set as the second to fourth *z* coordinate above the detected cell base height. Typically, the third *z* coordinate above the cell base is the maximum intensity of the *z* plane. The mean intensity is then calculated across all such representative pixels. Each channel is then normalized by the respective mean intensity in that cell.

#### Stress granule detection

To analyse the interactions of stress granules with their surrounding, we need to first identify the granules. Stress granules were detected in the G3BP1 channel using an automated routine probing various global thresholds. This routine yields the spatial coordinates, centroid, volume and eccentricity of well-defined stress granules within the bulk of the cytoplasm.

In the first step, the G3BP1 channel is filtered by subtracting a dynamic background. The background is determined by convolving a 30 × 30 × 5 box kernel on the confocal stack. The filtered image is then slightly blurred using the imgaussfilt command (MATLAB) to decrease shot noise. The lowest-probed threshold was set as the maximum of the mean intensity values in the *x*–*y* plane of the filtered G3BP1 channel across the height of the stack. The maximal threshold was set as the global maximum. Then, 101 threshold values spaced evenly over the resulting range have been probed. For each threshold, the regionprops3 function (MATLAB) has been used to detect volumetric blobs with intensity values above the threshold. The number of detected blobs as well as median and mean volume have been recorded for each threshold. The aim is to detect stress granules, that is, a group of finite size consisting of neighbouring voxels. Further, the group of voxels must have higher intensity compared with the environment and be set apart by a reasonably sharp gradient in intensity to ensure that we do not detect fluctuations in the diffuse G3BP1 protein. Consequently, the optimal threshold shows a minimal change in the number of detected blobs on varying the threshold level and corresponds to a large median and mean volume, that is, all or most of the granules in the cell are captured. The mean volume ensures that we detect a large portion of the granules and do not overestimate the threshold. The median volume is sensitive to the distribution of sizes. Thresholds that are too low typically yield one large detected volume and many small blobs on the periphery. Thresholds that yield many small blobs will have a low median volume. The median volume consequently offers a handle to avoid underestimating the threshold. Weighting these three criteria (namely, change in number of granules, mean volume and median volume) allows to assign a quality factor to each threshold and to detect a suitable value. Cells that do not exceed a minimal quality factor for the stress granule detection were discarded. In the second step, a logical three-dimensional mask of the detected granules, that is, false where there is no granule and true for all the voxels that are a part of the granule, has been inflated by dilation with a sphere having a radius of 3 pixels. Applying these granule masks on the initial G3BP1 confocal stack, we can calculate the weighted centroid of the inflated and initially detected blobs. If the weighted centroids of a given blob differed by more than 0.5 pixels, the blob has also been discarded to ensure robustness of detection. Moreover, all the pairs of granules that fused on dilation have been discarded to ensure a minimal distance between the granules. Moreover, granules, typically very small, that reside below or above the cell nucleus were discarded as they fall outside the cell mask. The ellipticity of each granule has been defined as the ratio of the major principal axis to minor principal axis of an ellipse fitted to each granule. Here we only considered the principal axes in the *x*–*y* plane. The effective radius of the granule is defined as ${R}_{{{\mathrm{SG}}}}=\sqrt{{A}_{{{\mathrm{SG}}}}/\uppi },$ where *A*_SG_ is the area of the stress granule projected onto the *x*–*y* plane like a cast shadow.

Aside from the characteristics (position, orientation, volume and so on), a number of images are saved for each granule. All the images are centred on the centroid of a given granule and show the *x*–*y* plane closest to the granule centroid. Note that the pixels within these images that fall outside the cell limits or inside the respective cell nucleus are set to NaN. The size of these images is 221 pixels × 221 pixels (about 14 µm ×14 μm) to also capture the long-range deformations of the tubulin network around the granules.

In the following analysis, granules with a radius below 3 pixels are discarded. Therefore, only granules with a diameter larger than twice the theoretical diffraction limit of 130 nm are considered (‘Cell imaging’ section).

In the final step, we filter the granules after calculation of the individual distribution map in the G3BP1 channel (Fig. 1i). Granules that are not at least twice as bright as their local environment are discarded. The local environment is defined by dilating the individual granule shape with a disc having a radius of 8 pixels and subtracting the original granule shape. Further, granules with a mean relative intensity higher than the mean plus one standard deviation of the relative intensity across all the granules are discarded to account for positive outliers.

#### Construction of the reference cell

The alignment of all the cells in three dimensions allows to construct cell stacks by overlaying all the cells that belong to the same experimental conditions, namely, the duration of arsenite treatment. Each cell stack is blurred in the *x*–*y* plane using a Gaussian kernel with a variance of 4 pixels. Although this method retains the overall intensity, noise as well as single filaments are blurred to suppress short-range fluctuations. To capture the finite size of the cytosol as well as of the nucleus, each cell is masked by the cell outline and nucleus shape. Based on these stacks, the reference intensity for any spatial coordinate is calculated as the mean intensity across all the cells at this position, omitting the data from cells where the given pixel is masked. If more than half the intensity values for a given coordinate fall within these masks of the nucleus or cell outline, the pixel is considered outside the cytoplasm of the reference cell. This way, the reference cell also serves as a mask for the expected cell shape (Fig. 1g,h).

Note that the symmetry along the short axis has been deliberately broken by rotating all the cells such that the cell nucleus falls into the same half of the cell. The remaining symmetry of the pattern allows to fold the cell along the centre line of the long axis to enhance the statistics for the calculation of the reference cell. Using *N* cells, this process yields 2*N* intensity values for all the spatial coordinates in one hemisphere of the cell. The full reference cell is then recovered by mirroring the result along the centre line.

Note that due to fluctuations in the height of the cells, we only considered the intensity values between the cell base height up to 1.2 μm (six slices) into the cell as reliable. Stress granules outside this range are not considered in the following analysis.

#### Correlation analysis

To calculate the averaged images, we first define a subset of granules with comparable size and ellipticity. Images of round granules (with a principal axis ratio not exceeding 1.5) are binned by the granule radius *R*_SG_ in steps of 3 pixels (195 nm). Given a minimal granule radius of 195 nm, the first size bin is [195, 325] nm. This size bin is then labelled as *R*_SG_ = 0.26 μm. Unless stated otherwise, data are shown in bins that are statistically independent, that is, each data point is assigned one bin. To calculate the averaged images of a given bin (Fig. 1e,f), we average the intensity values for a given pixel location from all the images of the G3BP1 and β-tubulin channel for granules of that bin. Note that we omit those pixels that fall outside the cytosol of the corresponding cell.

We calculate the distribution maps (Fig. 1k,l) in the same way as averaged images, except that each individual image is now normalized by the corresponding image of the reference cell. Normalization is done by point-wise division with the respective image originating from the reference cell of that channel at the same location. Note that if a pixel is masked in either reference or G3BP1 and β-tubulin image, it is considered masked. Consequently, different pixels of a distribution map may not have the same number of pixels that contributed to the calculation of the corresponding intensity value.

Fluctuations in the number of contributing datasets for different pixel locations introduce a nonlinearity when further analysing distribution maps, which has to be taken into account when calculating the radial distribution *g*(*r*) of a distribution map. To capture the varying statistical weight, we do not just take the radial average of the distribution map. Rather, we take the average over all the intensity values from the individually reference-cell-normalized images that the distribution map is calculated from at distance *r* from the centre, again omitting the masked entries. This way, we know how many data points contribute to each entry in *g*(*r*). The error of *g*(*r*) at a given distance *r* is then calculated as the standard error, that is, the standard deviation of the contributing values divided by the square root of the number of contributing values.

The surface-relative distribution function *g*_s_(*d*) is individually calculated from the reference-cell-normalized images for each granule, as it is the basis for the calculation of the partitioning coefficients for each granule. Here distance *d* for a given pixel location is calculated as the minimal distance to the outline of the stress granule. The outline itself has a value of *d* = 0 and pixels inside the granule have negative entries. The outline is defined by the stress granule detection routine. Then, *g*_s_(*d*) is calculated by averaging the intensities of pixels with the same *d*, omitting the masked pixels. The error is again calculated as the standard error.

#### Calculation of the partitioning coefficients

To calculate the partitioning coefficients *k*_g_ = *c*_g_/*c*_c_ and *k*_s_ = *c*_s_/*c*_c_, where *c*_g_, *c*_c_ and *c*_s_ are the β-tubulin concentrations inside the granule, in the cytosol and at the granule surface, respectively, we need to calculate the intensities of β-tubulin at the surface and in either bulk phase. Note that we assume that the intensity and concentration are linearly proportional. Given that tubulin shows little preference to either bulk phase and is enhanced by a factor of less than 1.2 at the interface compared with the cytosol, we assume the same proportionality constant in and around a granule, such that the proportionality constant cancels out when taking the ratio. To accurately assess the intensity at the interface and around it, we base our calculation on the surface-relative distribution function for β-tubulin *g*_s,TUB_(*d*). This way, variations in granule shape do not affect the measurement of surface intensity (Fig. 5a–c).

The intensity at the surface of a given granule *I*_s_ is calculated as the maximum intensity in *g*_s,TUB_(*d*) within –2 to +2 pixels (–0.13 to 0.13 μm) around the maximal gradient ∇*g*_s,SG_(*d*) of G3BP1, which corresponds to the position of the maximum adsorption in the limit of neutral wetting of tubulin particles to the surface. The interval is chosen to allow for variations in the peak position around the interface (Fig. 4c). At the surface, we find a broad peak of the tubulin intensity, with a width typically larger than the point spread function of the microscope (195 nm). Note that the apparent width of the peak coincides with the region over which the intensity of G3BP1 transitions from the bulk cytosol to bulk granule. To avoid artifacts from this broad peak across the interface of the stress granule, we measure the intensity inside the granule *I*_g_ as the mean of *g*_s,TUB_(*d*) inside the granule for *d* ≤ −0.39 μm from the detected surface. We also omit the entry in *g*_s,TUB_(*d*) with the largest distance from the surface, which corresponds to the very centre of the granule and typically has poor statistics. To have a reasonable set of pixels to average over, we only consider granules with a radius larger than 0.59 μm for this analysis, that is, at least three entries with *d* ≤ −0.39 μm.

We have previously shown that granules, also in nocodazole-treated cells, typically reside in a region of higher-than-average tubulin intensity (Fig. 1g,h and Supplementary Fig. 1). We can, therefore, not just take the expected tubulin intensity of the reference cell (which is one by construction) but also have to define an intensity for the surrounding of each granule *I*_c_. In nocodazole-treated cells, the long-range decay of tubulin around the granules is substantially less pronounced compared with cells having intact microtubule networks (Fig. 4), but still present. The intensity of the surrounding, thus, depends on the distance from the granule and is consequently subject to error. To capture the tubulin concentration that the granule experiences, we measure as close to the granule as possible and avoid artifacts from the broad surface peak in tubulin localized to the interface zone of the granule. We chose to define the intensity in the surrounding as the mean of the *g*_s,TUB_(*d*) between *d* = 0.39 and *d* = 0.52 μm outside the granule, keeping the same 0.39 μm away from the surface as for the calculation of *I*_g_. This half-width of the interface of 0.39 μm is chosen to be broad enough to also accommodate error from the position of the maximum gradient, which, on average, is found at *d* = 0.091 ± 0.007 μm (−1.4 ± 1.0 pixel) and thus does not coincide with *d* = 0.

#### Fitting

Fit parameters and errors are evaluated through variation of the fit over *N* = 1,000 realizations. From the initial fit of the theory (equation (5)) to the data, we extract the residuals and initial fit parameter *a*_0_. Using this fit parameter, we assign each data point with the theoretical prediction of −Δ*G*_s_ (−Δ*G*_g_) based on the −Δ*G*_g_ value of the data. For each iteration, we then add Gaussian noise to the data with zero mean and variance equal to the standard deviation of the residuals to create a variance of the original data and perform a new fit. The reported value of *a*_0_ is the mean of *N* = 1,000 realizations of the variation, with the error given as the standard deviation. All the fits use bisquare weighting of residuals.

#### Droplets in vitro

His- and GB1-tagged full-length FUS protein was purified from *Escherichia coli* under denaturing conditions with affinity chromatography. Overnight incubation with tobacco etch virus nuclear-inclusion-a endopeptidase (TEV protease) and subsequent second affinity column removed the tags. Protein was concentrated in the presence of 6 M urea up to 1.5 mM, similar to the FUS N-terminal domain (NTD) protocol^67^. To induce phase separation, the stock protein solution was diluted to a final concentration of 50 μM in a buffer (50 mM 4-(2-hydroxyethyl)-1-piperazineethanesulfonic acid (HEPES), 150 mM NaCl, pH 7.5). RNA was added to the FUS solution in the form of poly-U (Sigma-Aldrich) at a final concentration of 0.5 mg ml^–1^. To image the droplets in fluorescence, 1 μM Alexa Fluor 647 (ThermoFisher Scientific) was mixed into the droplet solution, where it partitioned into the condensed phase.

Then, 10% rhodamine-tagged porcine tubulin (Cytoskeleton) was mixed with purified bovine tubulin in tubulin buffer (80 mM piperazine-*N*,*N*′-bis(2-ethanesulfonic acid) (PIPES), 1 mM ethylene glycol-bis(β-aminoethyl ether)-*N*,*N*,*N*′,*N*′-tetraacetic acid (EGTA), 2 mM MgCl_2_, pH 6.9). This was either added to the droplets as sub-units at a final concentration of 0.1 mg ml^–1^, or first polymerized in the presence of 1 mM GTP and stabilized with Taxol (Paclitaxel, ThermoFisher Scientific). A small amount of the resulting microtubules were then added to the droplet solution.

Directly after mixing, the droplets were pipetted between sandwiched coverslips containing a coating of oil with 2% FluoroSurfactant (RAN Biotechnologies). The coverslips were sealed and imaged with ×60 water-immersion lens (NA = 1.2) on the same confocal microscope used to record the cell stacks.

## Online content

Any methods, additional references, Nature Research reporting summaries, source data, extended data, supplementary information, acknowledgements, peer review information; details of author contributions and competing interests; and statements of data and code availability are available at 10.1038/s41567-022-01537-8.

## Supplementary information

Supplementary Information Supplementary Figs. 1–9 and Sections 1–4.

Supplementary Video Epifluorescence time lapse of arsenite-treated U2OS cells. Tubulin is shown in green and G3BP1 is shown in magenta. The field of view is 26.3 × 16.4 μm^2^. The time stamp is in mm:ss.

## Data Availability

Source data are provided with this paper. The datasets generated and/or analysed during the current study are available at https://www.research-collection.ethz.ch (ref. ^68^).

## Source data

Source Data Fig. 1 Scatter plot of the bulk and surface affinities shown in Fig. 5.

## Code availability

The code developed and used in the current study is available at 10.5905/ethz-1007-484. This code, together with the supplied data, reproduces the relevant calculations as well as figures shown throughout the main text and Supplementary Information.
