# Publisher Correction: Non-specific adhesive forces between filaments and membraneless organelles

**DOI:** 10.1038/s41567-022-01600-4

**Published:** 2022-04-01

**Authors:** Thomas J. Böddeker, Kathryn A. Rosowski, Doris Berchtold, Leonidas Emmanouilidis, Yaning Han, Frédéric H. T. Allain, Robert W. Style, Lucas Pelkmans, Eric R. Dufresne

**Affiliations:** 1 Department of Materials, ETH Zurich, Zurich, Switzerland; 2 Department of Molecular Life Sciences, University of Zurich, Zurich, Switzerland; 3 Institute of Biochemistry, ETH Zurich, Zurich, Switzerland

**Keywords:** Biopolymers in vivo, Wetting, Polymers, Colloids, Nanoscale biophysics

Correction to: *Nature Physics* 10.1038/s41567-022-01537-8, published online 24 March 2022.

In the version of this article initially published, there was a labeling mistake in the Fig. 2d *y*-axis color bar units. Now showing a range from “0” to “6.0,” the original upper label was “1.2.” The error has been corrected in the HTML and PDF versions of the article.

