# Supplementary material for: Non-specific adhesive forces between filaments and membraneless organelles
Source: Nat Phys. 2022 Mar 24;18(5):571–8. doi: 10.1038/s41567-022-01537-8 (PMC9106579; doi:10.1038/s41567-022-01537-8)
Supplement: Supplementary file 1 — Supplementary Figs. 1–9 and Sections 1–4. [file 41567_2022_1537_MOESM1_ESM.pdf]

nature portfolio

https://doi.org/10.1038/s41567-022-01537-8

Supplementary information

Non-specific adhesive forces between filaments and membraneless organelles

In the format provided by the authors and unedited

Supplement: Non-specific adhesive forces between filaments and membraneless organelles

Thomas J. Böddeker,[1] Kathryn A. Rosowski,[1] Doris Berchtold,[2]
Leonidas Emmanouilidis,[3] Yaning Han,[3] Frédéric H. T. Allain,[3]
Robert W. Style,[1] Lucas Pelkmans,[2] and Eric R. Dufresne [a1]

[1]*Department of Materials, ETH Zurich, Zurich, Switzerland*
[2]*Department of Molecular Life Sciences, University of Zurich, Zurich, Switzerland*
[3]*Institute of Biochemistry, ETH Zurich, Switzerland*

(Dated: February 28, 2022)

[a] corresponding author eric.dufresne@mat.ethz.ch

I. SUPPLEMENTARY MOVIE

Epifluorescence time lapse of arsenite-treated U2OS cells. Tubulin in green and G3BP1 in magenta. Field of view is $26.3 \times 16.4\,\mu m$. Time stamp is in mm:ss.

II. SUPPLEMENTARY FIGURES

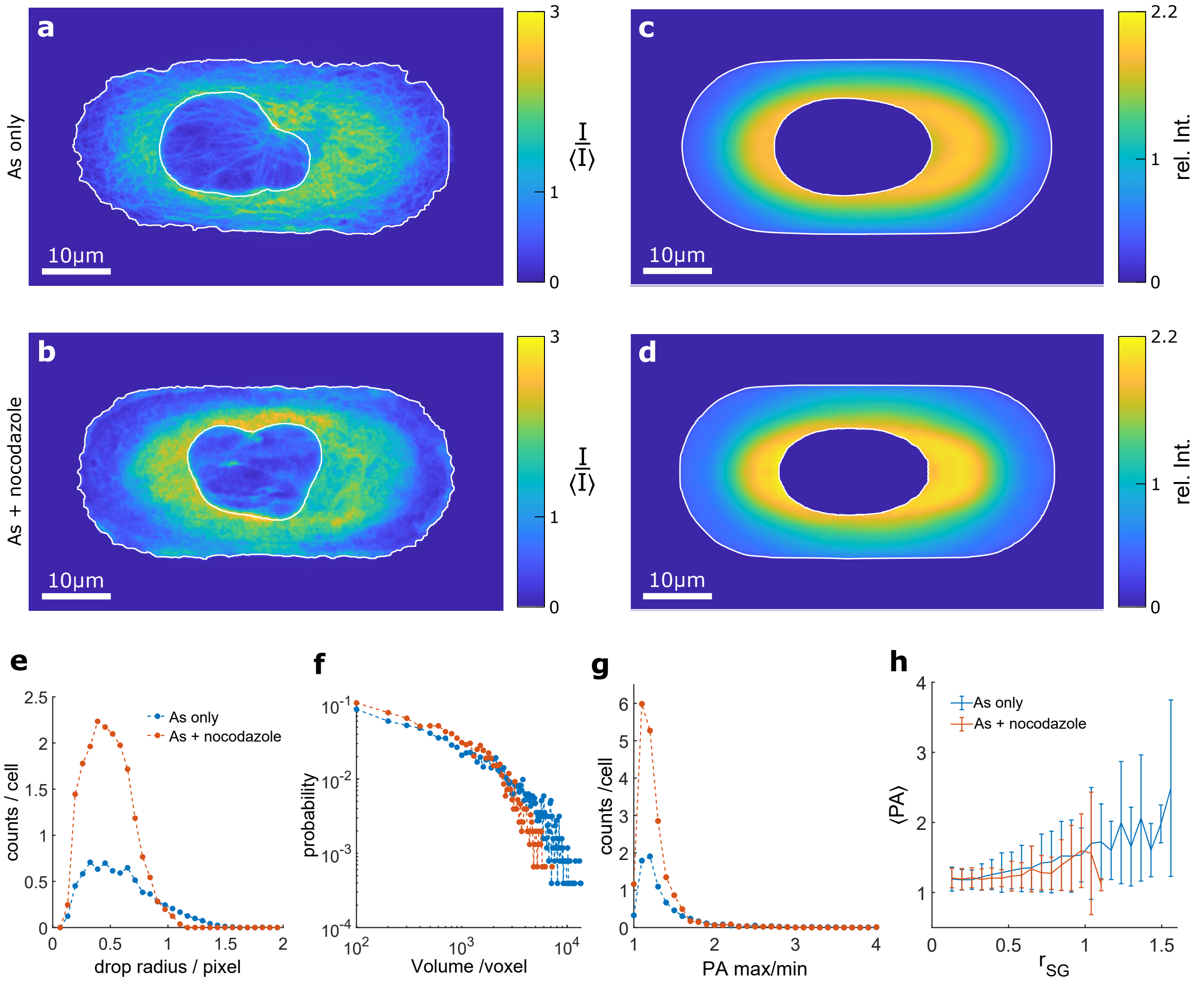

FIG. S1. Comparison of cells with and without nocodazole treatment **a**, xy-slice of the tubulin channel of an exemplary cell with intact microtubule network. Cell and nucleus outline are shown in white. **b**, xy-slice of the tubulin channel of an exemplary cell after 90 minutes of nocodazole treatmeant. Cell and nucleus outline are shown in white. **c**, xy-slice of the tubulin reference cell with intact microtubule network. Average cell and nucleus outline are shown in white. **d**, xy-slice of the tubulin reference cell after 90 minutes of nocodazole treatmeant. Average cell and nucleus outline are shown in white. **e**, Histogram of observed granule radii with a total of 2543 granules from 335 cells with intact microtubule network and 1520 granules from 81 cells with nocodazole treatment. **f**, Histogram of granule volume. **g**, Histogram of the principal axis ratio. **h**, Mean principal axis ratio as a function of granule radius. The errorbars are given as the standard deviation.

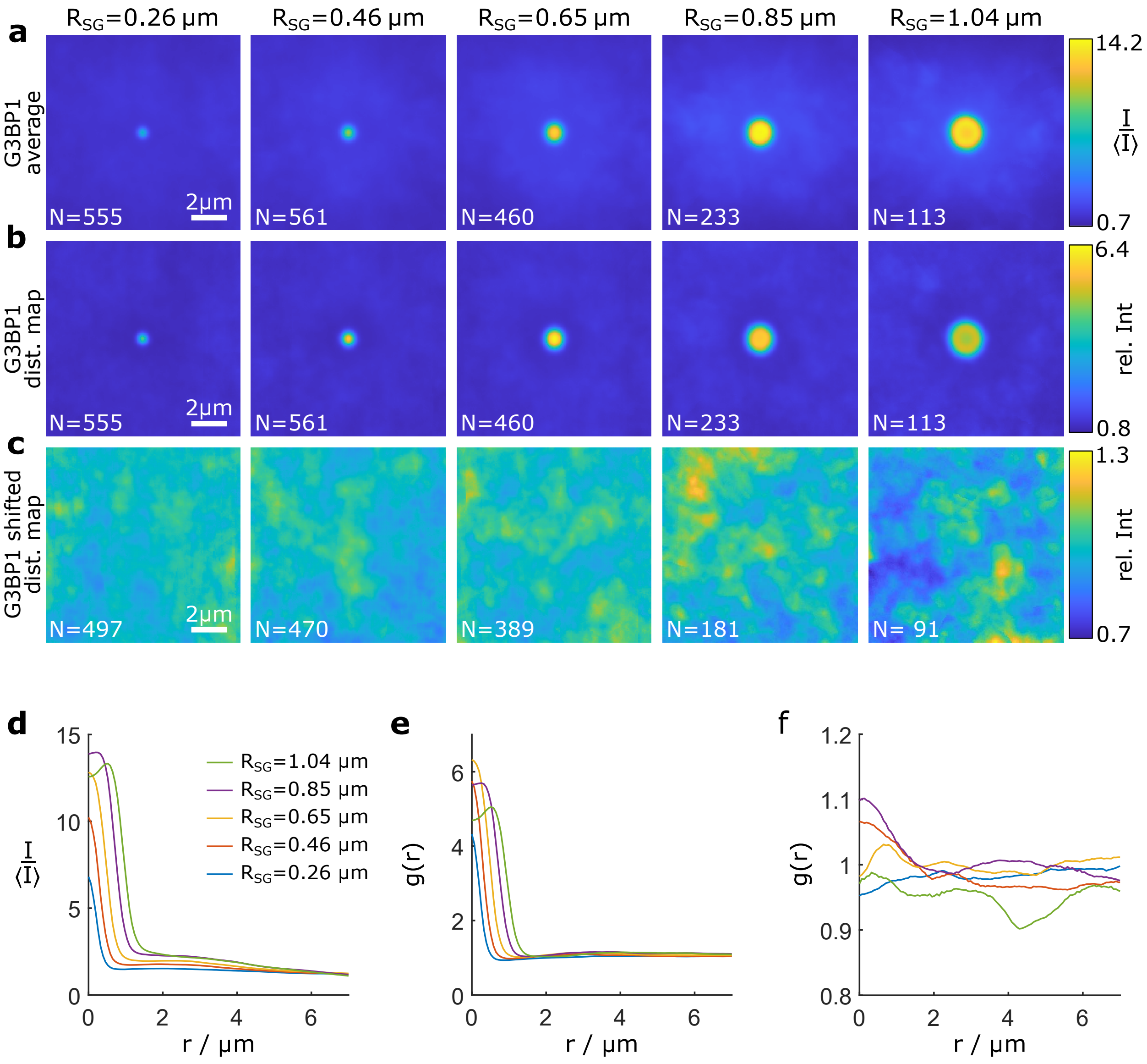

FIG. S2. Average images, and distribution maps of stress granules. **a**, Average images of G3BP1 for different granule sizes, N gives the number of contributing granules. **b**, Distribution maps of G3BP1. **c**, Negative control: Distribution maps of G3BP1 where images of granule in cell i have been extracted at the same location in cell $i+1$, probing random but biologically plausible input. Note that granules with locations that fall within the cell nucleus or outside the cell at cell $i+1$ are discarded. **d**, Radial curves corresponding to the average images in panel **a**. **e**, $g(r)$ corresponding to the distribution maps in panel **b**. **f**, $g(r)$ corresponding to the distribution maps in panel **c**. Note that we do not show standard error here because it is smaller than the fluctuations seen in $g(r)$ of the shifted images.

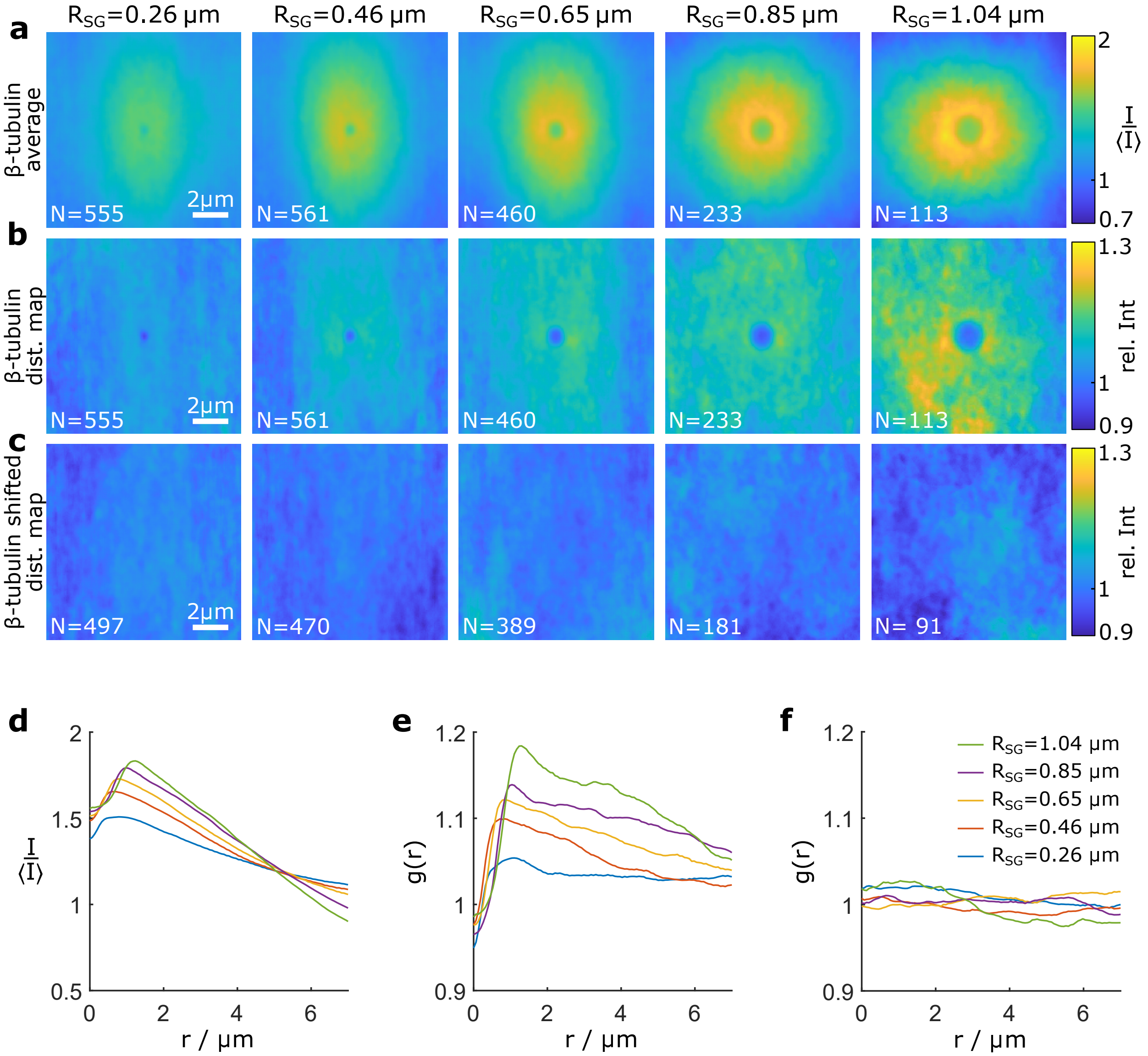

FIG. S3. Average images, and distribution maps of β-tubulin. **a**, Average images of β-tubulin for different granule sizes, N gives the number of contributing granules. **b**, Distribution maps of β-tubulin. **c**, Negative control: Distribution maps of β-tubulin where images of the β-tubulin channel in cell i have been extracted at the same location in cell $i + 1$, probing random but biologically plausible input. Note that images with locations that fall within the cell nucleus or outside the cell at cell $i + 1$ are discarded. **d**, Radial curves corresponding to the average images in panel **a**. **e**, $g(r)$ corresponding to the distribution maps in panel **b**. **f**, $g(r)$ corresponding to the distribution maps in panel **c**.

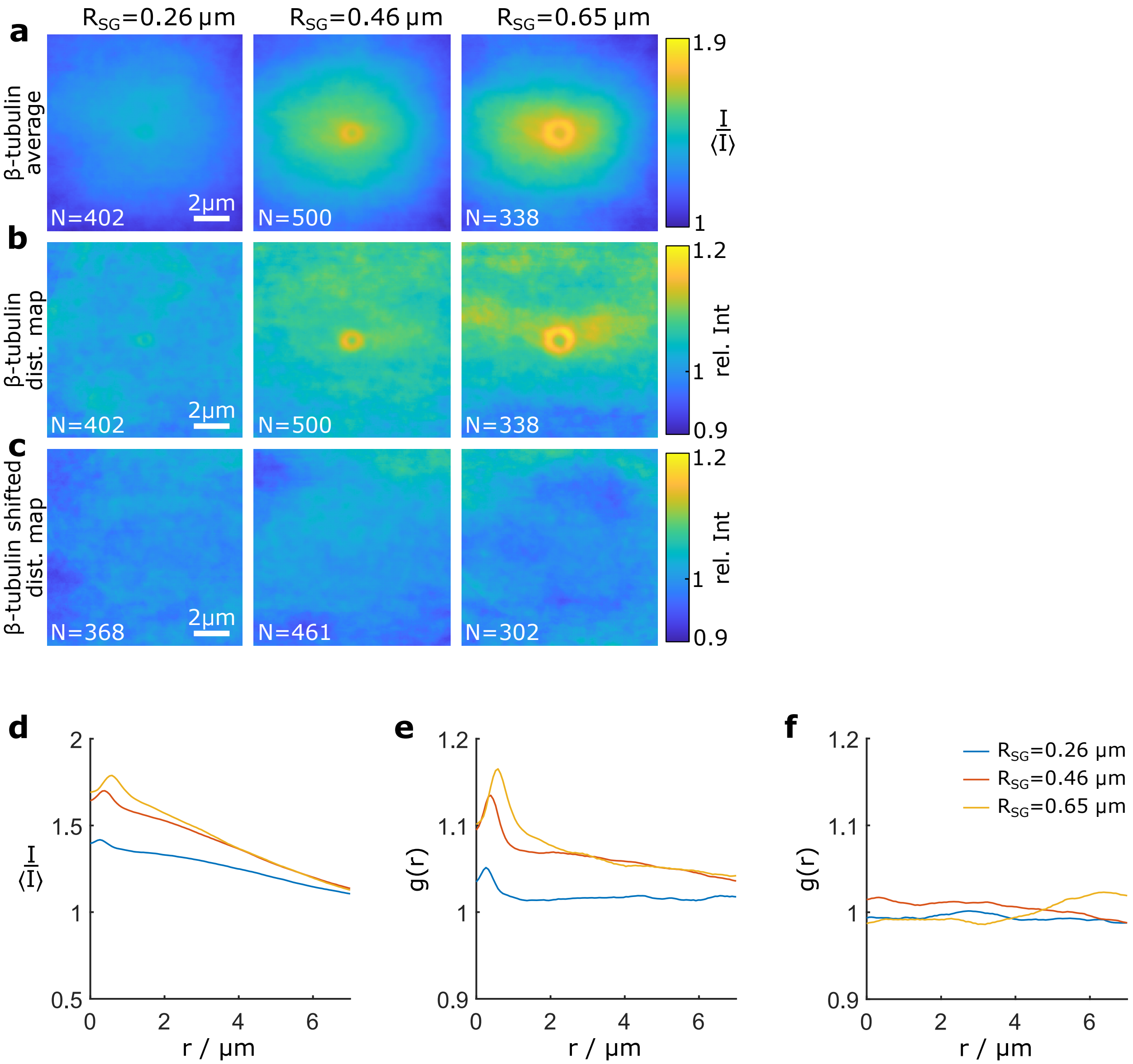

FIG. S4. Average images, and distribution maps of β-tubulin in nocodazole-treated cells. **a**, Average images of β-tubulin for different granule sizes, N gives the number of contributing granules originating from nocodazole-treated cells. **b**, Distribution maps of β-tubulin in nocodazole-treated cells. **c**, Negative control: Distribution maps of β-tubulin where images of the β-tubulin channel in cell i have been extracted at the same location in cell $i+1$, probing random but biologically plausible input from nocodazole-treated cells. Note that images with locations that fall within the cell nucleus or outside the cell at cell $i+1$ are discarded. **d**, Radial curves corresponding to the average images in panel **a**. **e**, $g(r)$ corresponding to the distribution maps in panel **b**. **f**, $g(r)$ corresponding to the distribution maps in panel **c**.

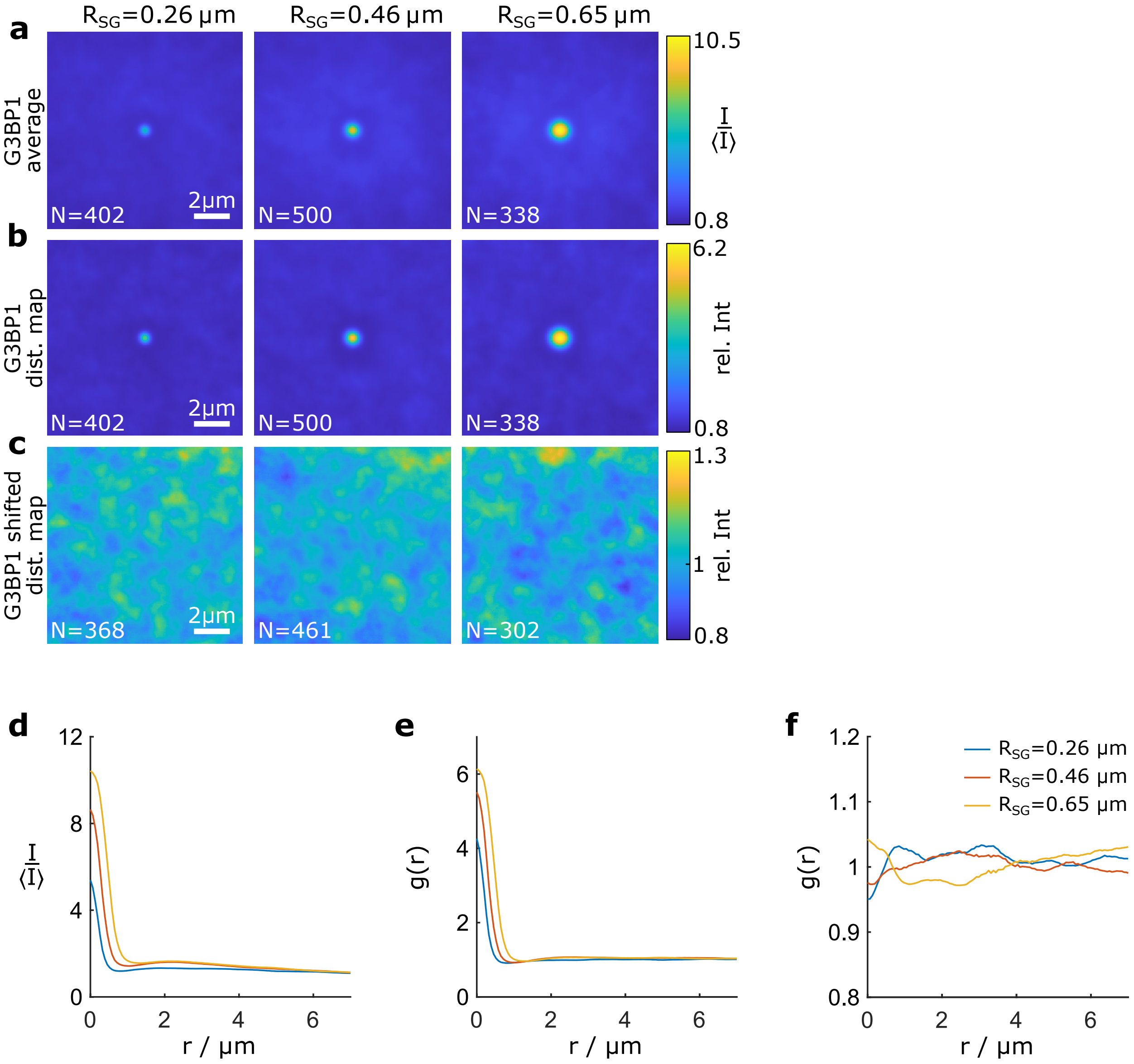

FIG. S5. Average images, and distribution maps of G3BP1 in nocodazole-treated cells. **a**, Average images of G3BP1 for different granule sizes, N gives the number of contributing granules originating from nocodazole-treated cells. **b**, Distribution maps of G3BP1 in nocodazole-treated cells. **c**, Negative control: Distribution maps of G3BP1 where images of the β-tubulin channel in cell i have been extracted at the same location in cell $i+1$, probing random but biologically plausible input from nocodazole-treated cells. Note that images with locations that fall within the cell nucleus or outside the cell at cell $i+1$ are discarded. **d**, Radial curves corresponding to the average images in panel **a**. **e**, $g(r)$ corresponding to the distribution maps in panel **b**. **f**, $g(r)$ corresponding to the distribution maps in panel **c**.

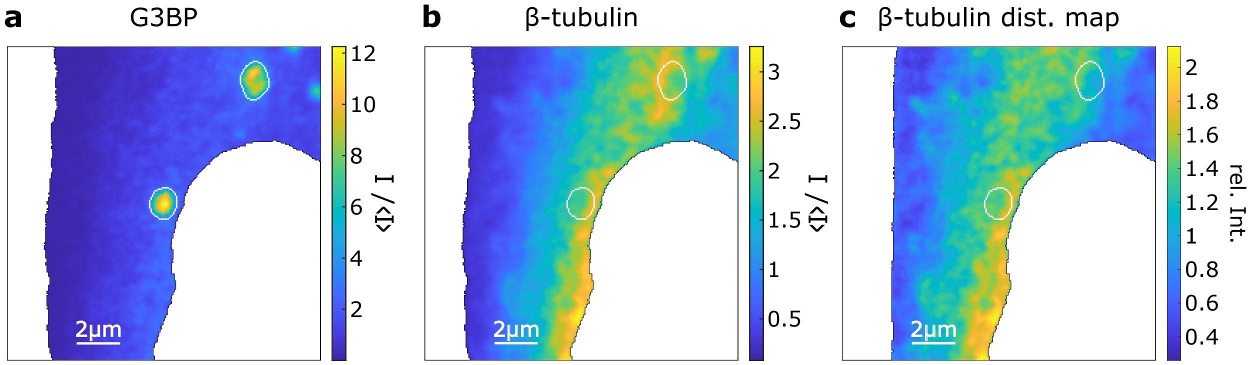

FIG. S6. Exemplary image of stress granules in cells after 90 minutes of nocodazole treatment. **a**, G3BP channel centered on the stress granule of which the surface-relative distribution function is shown in Fig. 5 **a**. The outline of this granule and a second large one are indicated in white. **b**, Tubulin channel at the same location. **c**, Distribution map of tubulin at the same location.

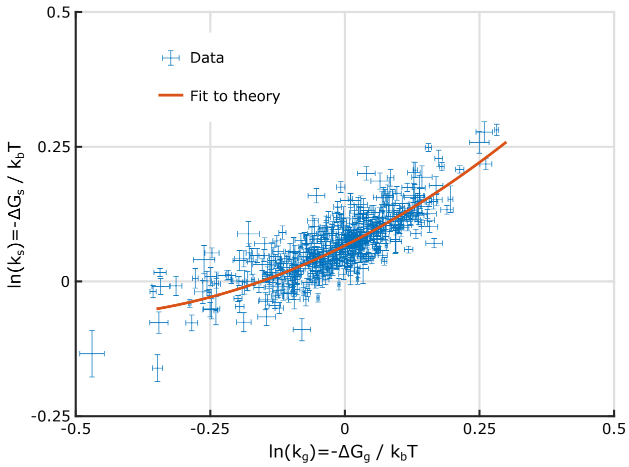

FIG. S7. Scatter of $\ln(k_s)$ as a function of $\ln(k_g)$ as shown in Fig. 5 **e** with error bars. Error bars are calculated through error propagation of the standard errors of the respective experimental measurements.

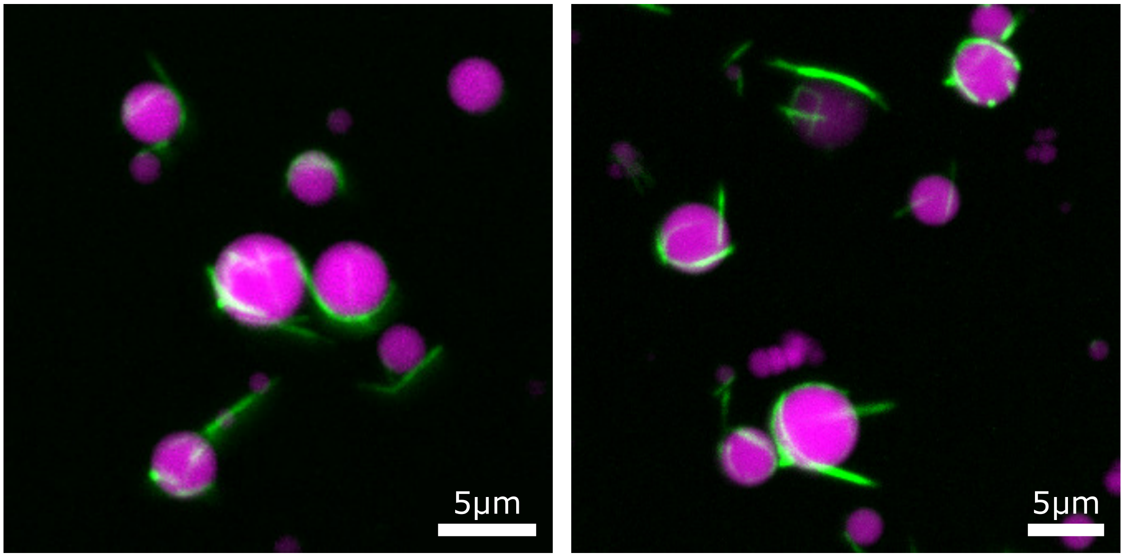

FIG. S8. FUS-RNA droplets (magenta) with taxol-stabilized microtubules (green). Identical experimental conditions as in Fig. 6 **e-i**.

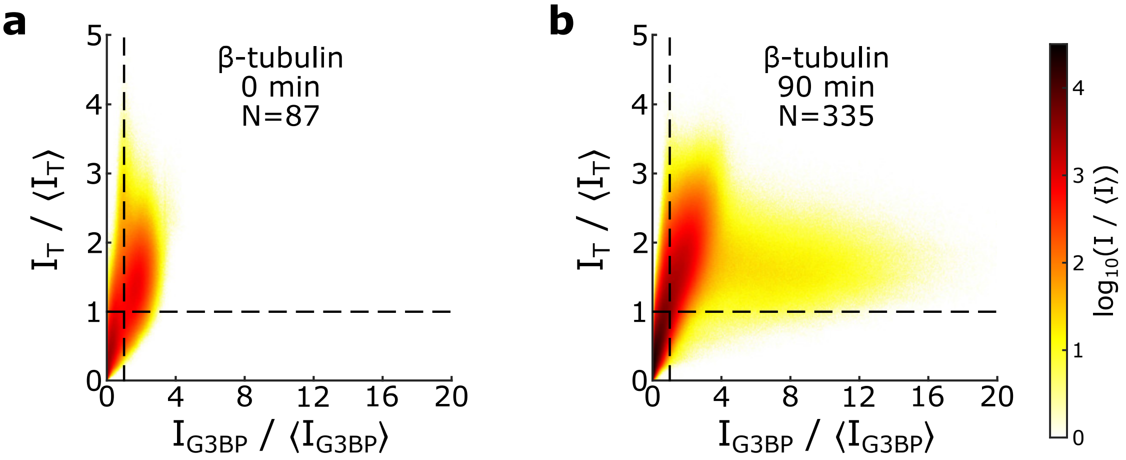

FIG. S9. Spatial correlation between tubulin and G3BP1 in the reference cells. **a**, For cells without arsenite treatment (0 minutes of arsenite treatment). **b**, After 90 minutes of arsenite treatment. ⟨·⟩ gives the average intensity of the respective channel.

III. THEORY

A. Partition Coefficients

To evaluate under which conditions a tubulin sub-unit will prefer to reside on the granule surface or in bulk cytosol or bulk granule, we identify the chemical potential μ for each state assuming tubulin is dilute:

$$\mu_c = \mu_c(c_0, T) + k_b T \ln\left(\frac{c_c}{c_0}\right) \tag{1}$$

$$\mu_g = \mu_g(c_0, T) + k_b T \ln\left(\frac{c_g}{c_0}\right) \tag{2}$$

$$\mu_s = \mu_s(c_0, T) + k_b T \ln\left(\frac{c_s}{c_0}\right), \tag{3}$$

where c_0 is an arbitrary reference concentration. Note that the surface concentration c_s is also a volumetric concentration. This is becausewe measure intensity per voxel in the experiment. Moreover, the assumption that tubulin is dilute implies that tubulin sub-units do not feel and interact with each other.

We assume a local equilibrium of the chemical potential of tubulin in and around the granule. We justify this for once, because we consider a small subset of the cell, i.e. inside and outside of the granule are close together (about 1 µm) such that we expect diffusion to relax gradients in chemical potential, and because we assume that no chemical reactions occur as tubulin moves from one phase to the other. Equating the chemical potentials of a sub-unit in the cytosol and inside the granule, we can define the partition coefficient between granule and cytosol

$$k_g = \frac{c_g}{c_c} = \exp\left(\frac{-\Delta G_g}{k_b T}\right), \tag{4}$$

with affinity for the bulk of the granule $-\Delta G_g = \mu_c(c_0, T) - \mu_g(c_0, T)$ between a sub-unit in the granule compared to the cytosol. Analogously, we define the partitioning coefficient to the surface relative to the cytosol as

$$k_s = \frac{c_s}{c_c} = \exp\left(\frac{-\Delta G_s}{k_b T}\right), \tag{5}$$

with surface affinity $-\Delta G_s = \mu_c(c_0, T) - \mu_s(c_0, T)$.

In order to calculate the free energy differences ΔG_g and ΔG_s, we present two models, which differ in the definition of the interface between stress granule and cytosol. The tubulin sub-unit is, in both cases modeled as a colloidal particle.

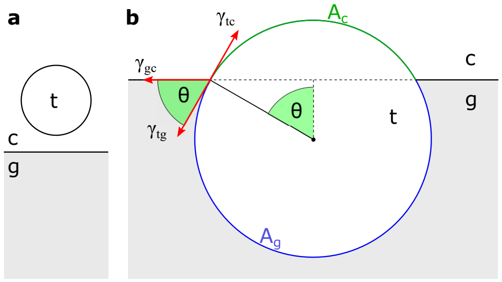

FIG. S10. Tubulin sub-unit modelled as a colloidal particle t interacting with a thin interface between stress granule g and cytosol c. **a**, Reference state of a sub-unit with surface area A_0 in bulk cytosol. **b**, A sub-unit wetting the interface with contact angle θ. A_c is the surface area of the spherical cap of the sub-unit exposed to the cytosol, $A_g = A_0 - A_c$ respectively the spherical cap exposed to the granule.

B. Thin interface

First, let us consider the colloidal model tubulin particle of radius R interacting with an interface of width $2w$ that is much thinner than the size of the particle $R >> w$. (Note that the smallest possible tubulin particle is a sub-unit but we cannot ensure that all tubulin particles are sub-units in nocodazole-treated cells.) This corresponds to the classical picture of a particle at an interface as shown in Fig. S10. $-\Delta G_g = \mu_c(c_0, T) - \mu_g(c_0, T)$ is the affinity of a sub-unit towards the bulk phase, i.e. the energy difference a sub-unit experiences when it is moved from bulk cytosol to inside the granule, where a positive value means that the inside of the granule is energetically favorable. $-\Delta G_g$ is then given as the surface area of the sub-unit A_0 times the difference in the surface tension of the sub-unit towards the cytosol γ_{tc} and towards the bulk of the granule γ_{tg}

$$-\Delta G_g = A_0(\gamma_{tc} - \gamma_{tg}). \tag{6}$$

In order to calculate the surface affinity $-\Delta G_s = \mu_c(c_0, T) - \mu_s(c_0, T)$ between a sub-unit in bulk cytosol and adhered to the surface of the granule, let us first consider the energy of a sub-unit at the interface. This energy is given as the balance of the area of the interface taken up by the sub-unit A_x times the surface tension of the granule towards the cytosol γ_{cg} and the surface areas A_c and $A_0 - A_c$ of the granule exposed to the cytosol and the granule

multiplied with the respective surface tension:

$$\mu_s(c_0, T) = A_c\gamma_{tc} + (A_0 - A_c)\gamma_{tg} - A_x\gamma_{cg}.$$

The surface affinity is then

$$\begin{aligned} -\Delta G_s &= \mu_c(c_0, T) - \mu_s(c_0, T) \\ &= A_0\gamma_{tc} - (A_c\gamma_{tc} + (A_0 - A_c)\gamma_{tg} - A_x\gamma_{cg}) \\ &= (A_0 - A_c)\gamma_{tc} - (A_0 - A_c)\gamma_{tg} + A_x\gamma_{cg}. \end{aligned} \tag{7}$$

Using Young's law

$$\gamma_{tc} = \gamma_{tg} + \gamma_{cg}\cos(\theta), \tag{8}$$

with contact angle θ, we find

$$\begin{aligned} -\Delta G_s &= (A_0 - A_c)(\gamma_{tg} + \gamma_{cg}\cos(\theta)) \\ &\quad - (A_0 - A_c)\gamma_{tg} + A_x\gamma_{cg} \\ &= (A_0 - A_c)\gamma_{cg}\cos(\theta) + A_x\gamma_{cg}. \end{aligned} \tag{9}$$

To define the geometric quantities, we now consider a spherical sub-unit, as illustraded in Fig. S10. The sub-unit with radius R then has a surface area $A_0 = 4\pi R^2$, surface area exposed to the cytosol (in the shape of a spherical cap) of $A_c = 2\pi R^2(1 - \cos(\theta))$ and an area $A_x = \pi R^2 \sin^2(\theta)$ that the sub-unit takes up on the surface of the granule, with contact angle θ as defined in Fig. S10 **b**. Thus we finally arrive at

$$\begin{aligned} -\Delta G_s &= \left(4\pi R^2 - 2\pi R^2(1 - \cos(\theta)\right)\gamma_{cg}\cos(\theta) \\ &\quad + \pi R^2\gamma_{cg}\sin^2(\theta) \\ &= \pi R^2\gamma_{cg}\left(2\cos^2(\theta) + \sin^2(\theta) + 2\cos(\theta)\right) \\ &= \pi R^2\gamma_{cg}\left(1 + \cos(\theta)\right)^2. \end{aligned} \tag{10}$$

The energy difference between a particle in bulk cytosol compared to bulk granule is then analogously

$$\begin{aligned} -\Delta G_g &= 4\pi R^2(\gamma_{tc} - \gamma_{tg}) \\ &= 4\pi R^2\gamma_{cg}\cos(\theta). \end{aligned} \tag{11}$$

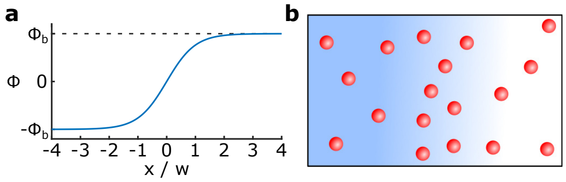

FIG. S11. Tubulin sub-units interacting with an extended interface between granule and cytosol. **a**, Phases and interface are determined by $\phi(x) = \phi_b \tanh\left(\frac{x}{w}\right)$. **b** Tubulin sub-units (red) are smaller than the interface width $2w$.

For comparison to our data, we express ΔG_s in terms of ΔG_g

$$-\Delta G_s = \pi R^2 \gamma_{cg} + \frac{-\Delta G_g}{2} + \frac{(-\Delta G_g)^2}{16\pi R^2 \gamma_{cg}}. \tag{12}$$

Note that this expression has removed the explicit dependence on contact angle. $-\Delta G_g$ is a function of both γ_{cg} and θ. The histogram of observed $-\Delta G_g$ (Fig. 5 (**d**)), however, shows both positive and negative values with a mean close to zero. If θ was constant, this would call for negative surface tension γ_{cg}, which is not physical. We thus assume that variations in $-\Delta G_g$ are dominated by variations in contact angle θ and hold γ_{cg} constant.

C. Thick interface

Tubulin sub-units, as well as the constituents of stress granules, are proteins, i.e. the assumption that the particle interacting with a droplet interface is much larger than the interface width is not given. To set up a theory for a thick interface ($R \ll w$), we follow the approach presented by Cahn and Hillard [1]. We consider a two-phase system characterized by an intensive scalar quantity ϕ (other than temperature or pressure) that transitions smoothly from one state (inside the bulk of the stress granule) to another state (bulk cytosol). Here we express ϕ as the local mesoscale composition

$$\phi(x) = \langle 1 - 2\psi_g(x) \rangle_{meso}, \tag{13}$$

where ψ_g gives the local volume fraction of stress granule components and $\langle \cdot \rangle_{meso}$ gives the local average over a volume sufficiently large such that ϕ is smooth [2]. The spatial coordiante x is normal to a flat interface between granule and cytosol, with the midpoint of the interface at $x = 0$, without loss of generality.

We assume a Helmholtz free energy per unit volume of the system given as

$$f(\phi, \nabla\phi) = \frac{a}{2}\phi^2 + \frac{b}{4}\phi^4 + \frac{\kappa}{2}(\nabla\phi)^2, \tag{14}$$

with parameter $a = a(T)$ that is negative under conditions in which the system separates and positive constants b and κ [1–3]. The volume terms capture the phase behavior of the system and the gradient term describes interfacial energies. For negative a, i.e. below the critical temperature, f has two minima at $\pm\phi_b = \sqrt{-a/b}$ [2]. Assuming a constant chemical potential μ everywhere in the system, it can be shown that the local composition takes the form [2, 3]

$$\phi(x) = \phi_b \tanh\left(\frac{x}{w}\right), \tag{15}$$

with width of the interface [3]

$$w = \sqrt{\frac{-2\kappa}{a}}. \tag{16}$$

Integrating the free energy over the interface, one finds the surface tension between both phases [2]

$$\gamma_{cg} = \frac{2a^2 w}{3b}. \tag{17}$$

To evaluate the energy per unit volume around the interface, we expand $\phi(x)$ around $x = 0$

$$\begin{aligned} \phi(x) &= \phi_b \left(\frac{x}{w} - \frac{x^3}{3w^3} + \mathcal{O}(x^4)\right) \\ \nabla\phi(x) &= \phi_b \left(\frac{1}{w} - \frac{x^2}{w^3} + \mathcal{O}(x^3)\right). \end{aligned} \tag{18}$$

Inserting into equation 14 we find

$$f(x) \approx \frac{3\gamma_{cg}}{2w}\left(\frac{1}{4} - \frac{x^2}{w^2}\right). \tag{19}$$

Far from the interface, we find for either bulk phase

$$f(\pm\infty) = \frac{-3\gamma_{cg}}{8w}. \tag{20}$$

Moving a particle with surface area A_0 and volume V_0 from bulk cytosol ($x \to \infty$) to position x we then change the free energy by

$$\Delta G(x) = A_0\,(\gamma(x) - \gamma(\infty)) - V_0\,(f(x) - f(\infty)). \tag{21}$$

$\Delta G(x)$ consists of a contribution from the surface tension of the particle proportional to its surface area and a term that captures the energy of the displaced fluid. For the change in surface tension we apply the law of mixtures and find

$$\gamma(x) - \gamma(\infty) = \frac{\Delta\gamma}{2}\left(\frac{\phi_b - \phi}{\phi_b}\right) \approx \frac{\Delta\gamma}{2}\left(1 - \frac{x}{w}\right), \tag{22}$$

with $\Delta\gamma = \gamma_{tg} - \gamma_{tc}$, where the last term shows the expansion around $x = 0$. For the free energy difference between the bulk phases, the volume terms cancel out (see eq. 20) and we find

$$\Delta G_g = \Delta G(-\infty) = A_0 \Delta\gamma. \tag{23}$$

Inserting equations 19, 20 and 22 into equation 21, we can express the energy of a particle around the interface as

$$\Delta G(x) \approx A_0 \frac{\Delta\gamma}{2}\left(1 - \frac{x}{w}\right) - V_0 \frac{3}{2}\frac{\gamma_{cg}}{w}\left(\frac{1}{2} - \frac{x^2}{w^2}\right). \tag{24}$$

$\Delta G(x)$ has a minimum at the equilibrium position for the particle, for which we find

$$x_{eq} = \frac{A_0 w^2}{6V_0}\frac{\Delta\gamma}{\gamma_{cg}}, \tag{25}$$

with energy

$$\begin{aligned} \Delta G_s = \Delta G(x_{eq}) = &- \frac{3}{4}\frac{V_0\gamma_{cg}}{w} + \frac{1}{2}(A_0\Delta\gamma) \\ &- \frac{w}{24V_0\gamma_{cg}}(A_0\Delta\gamma)^2. \end{aligned} \tag{26}$$

Here we can identify ΔG_g and express the surface affinity $-\Delta G_s$ in terms of $-\Delta G_g$

$$-\Delta G_s = a_0 + \frac{1}{2}(-\Delta G_g) + \frac{(-\Delta G_g)^2}{32a_0}, \tag{27}$$

with parameter

$$a_0 = \frac{3}{4}\frac{\gamma_{cg}}{w}V_0 = \pi R^2 \gamma_{cg}\left(\frac{R}{w}\right), \tag{28}$$

assuming a spherical particle with radius R. Note that the theory predicts a broad adsorption peak with a width corresponding to the width of the interface.

IV. CALCULATION OF THE BENDOCAPILLARY LENGTH

To estimate the stress granule radius at which we can expect microtubules to bend around the granule we perform a gedankenexperiment where a microtubule wraps a stress granule once. The elastic energy to bend a length l of filament to a radius of curvature matching the radius of the stress granule R_{SG} is then

$$E_{bend}(l) = \int \frac{K_{MT}}{2R_{SG}^2} \mathrm{d}l = \frac{K_{MT}}{2R_{SG}^2} l,$$

with bending rigidity of the microtubule K_{MT} and line integral $\int \mathrm{d}l$. We can approximate K_{MT} from the persistence length l_p of microtubules, which we take to be about one millimeter [4]:

$$K_{MT} \approx l_p \cdot k_b T \approx 4 \cdot 10^{-24}\,\mathrm{Jm},$$

with thermal energy $k_b T$. The adhesion energy of a microtubule of length l in contact with the granule is then

$$E_{surf}(l) = W \cdot A_{contact} \approx \gamma_{cg}(1 + \cos(\theta)) \cdot l\, R_{MT},$$

with surface tension γ_{cg} between granule and cytosol, contact angle θ between granule and microtubule and microtubule radius R_{MT}. Typical values of the surface tension of protein droplets vary between 1 to $100\,\mu\mathrm{J/m^2}$ [5, 6]. Now we can define the bendocapillary length R_{bc} as the radius of a granule at which both forces are equal. For R_{bc} we find

$$R_{bc} \approx \sqrt{\frac{K_{MT}}{2R_{MT}\gamma_{cg}(1 + \cos(\Theta))}} \approx 4\,\mu\mathrm{m}$$

assuming neutral wetting, a surface tension of $10^{-5}\,\mathrm{J/m^2}$ and a microtubule radius of $R_{MT} \approx 12\,\mathrm{nm}$. Unconfined microtubules coming into contact with a stress granule with $R_{SG} \gtrsim R_{bc}$ are consequently expected to spontaneously wrap around the granule. Note that lower surface tension leads to larger R_{bc}.

V. REFERENCES

---

[1] J. W. Cahn and J. E. Hilliard, "Free Energy of a Nonuniform System. I. Interfacial Free Energy," *The Journal of Chemical Physics*, vol. 28, pp. 258–267, Feb. 1958.

[2] M. E. Cates and E. Tjhung, “Theories of Binary Fluid Mixtures: From Phase-Separation Kinetics to Active Emulsions,” *Journal of Fluid Mechanics*, vol. 836, p. P1, Feb. 2018.

[3] Y. J. Choi and P. D. Anderson, “Cahn-Hilliard modeling of particles suspended in two-phase flows: CAHN-HILLIARD MODELING OF PARTICLES SUSPENDED IN TWO-PHASE FLOWS,” *International Journal for Numerical Methods in Fluids*, vol. 69, pp. 995–1015, June 2012.

[4] F. Gittes, B. Mickey, J. Nettleton, and J. Howard, “Flexural rigidity of microtubules and actin filaments measured from thermal fluctuations in shape.,” *Journal of Cell Biology*, vol. 120, pp. 923–934, Feb. 1993.

[5] Y. Liu, R. Lipowsky, and R. Dimova, “Concentration Dependence of the Interfacial Tension for Aqueous Two-Phase Polymer Solutions of Dextran and Polyethylene Glycol,” *Langmuir*, vol. 28, pp. 3831–3839, Feb. 2012.

[6] M. Ijavi, R. W. Style, L. Emmanouilidis, A. Kumar, S. M. Meier, A. L. Torzynski, F. H. T. Allain, Y. Barral, M. O. Steinmetz, and E. R. Dufresne, “Surface tensiometry of phase separated protein and polymer droplets by the sessile drop method,” *Soft Matter*, vol. 17, no. 6, pp. 1655–1662, 2021.
